# Preterm and full term infant vocalization and the origin of language

**DOI:** 10.1038/s41598-019-51352-0

**Published:** 2019-10-14

**Authors:** D. Kimbrough Oller, Melinda Caskey, Hyunjoo Yoo, Edina R. Bene, Yuna Jhang, Chia-Cheng Lee, Dale D. Bowman, Helen L. Long, Eugene H. Buder, Betty Vohr

**Affiliations:** 1 University of Memphis, Memphis, Tennessee USA; 2 Institute for Intelligent Systems, University of Memphis, Memphis, Tennessee USA; 3 Konrad Lorenz Institute for Evolution and Cognition Research, Klosterneuburg, Austria; 4 Kaiser Permanente, Oregon, USA; 5 University of Alabama, Tuscaloosa, Alabama USA; 6 Chung Shan Medical University, Taichung, Taiwan; 7 Portland State University, Portland, Oregon, USA; 8 Alpert Medical School of Brown University, Women and Infants Hospital, Providence, RI USA

**Keywords:** Human behaviour, Evolutionary theory

## Abstract

How did vocal language originate? Before trying to determine how referential vocabulary or syntax may have arisen, it is critical to explain how ancient hominins began to produce vocalization flexibly, without binding to emotions or functions. A crucial factor in the vocal communicative split of hominins from the ape background may thus have been copious, functionally flexible vocalization, starting in infancy and continuing throughout life, long before there were more advanced linguistic features such as referential vocabulary. 2–3 month-old modern human infants produce “protophones”, including at least three types of functionally flexible non-cry precursors to speech rarely reported in other ape infants. But how early in life do protophones actually appear? We report that the most common protophone types emerge abundantly as early as vocalization can be observed in infancy, in preterm infants still in neonatal intensive care. Contrary to the expectation that cries are the predominant vocalizations of infancy, our all-day recordings showed that protophones occurred far more frequently than cries in both preterm and full-term infants. Protophones were not limited to interactive circumstances, but also occurred at high rates when infants were alone, indicating an endogenous inclination to vocalize exploratorily, perhaps the most fundamental capacity underlying vocal language.

## Introduction

In the origin of language, it appears increasingly probable that a crucial factor was copious, functionally flexible vocalization, starting in early infancy^1,2^. Without such vocal raw material, it seems vocal interaction leading toward speech and language could never have gotten off the ground. It has long been known that very young human infants produce “protophones”, including at least three types of functionally flexible non-cry precursors to speech^3,4^, reported to occur rarely in other ape infants^5^.

But how early in life do protophones actually appear? We report here on research with both preterm infants still in neonatal intensive care and full-term infants monitored at home from the first month after birth. Longitudinal all-day audio recordings were reviewed by trained human listeners for both groups of infants. The goal was to determine the extent to which protophones occurred in preterm infants along with cries and to provide a comparison of relative cry and protophone rates in both groups. It has often been thought that cries are the predominant vocalizations of early infancy and that protophones emerge from cries^6–8^. However, recent research has shown that protophones occur from the first days of life in full term infants and far more frequently than cries from at least 3 months of age^2,3,9^. The present work provides new perspectives on very early protophone and cry rates and even addresses the possibility that preterm infants, shortly after extubation, when they can first breathe independently, may also produce protophones and thus manifest a flexible vocalization capability required as a foundation for vocal language. Our method also allows evaluation of the extent to which protophones occur in both interactive and non-interactive circumstances and especially when infants are alone. That infants vocalize when they are not interacting with caregivers^10^ suggests an endogenous inclination to vocalize exploratorily, perhaps the most fundamental capacity underlying vocal language.

## Background on the Origins of Language

Roots of human speech and language are being sought in a variety of domains. Cross-species comparative evaluations have illustrated that many species have vocal capabilities that, while far less elaborate than those of humans, do display notable flexibility^11–14^. Apes have been shown to have important gestural communication abilities^15,16^, even in the first years of life^17,18^, and learning of considerable language-like behavior has been shown to be possible in animals trained by humans from an early age^19,20^. Computational modeling and robotics research has illustrated that aspects of language learning and evolution can be mutually informative and has encouraged the speculation that language evolution is driven by endogenous factors in infants, especially by curiosity^21–25^.

Human development research is central in the search for roots of language^26,27^, and our group has argued that longitudinal observation of human infants^2,28^ is especially important in providing evidence relevant to possible early changes in the hominin line that formed foundations for the evolution of language. This reasoning is based on the fact that human infants begin to talk only after crucial earlier steps of development that have been reasoned to have also been steps that ancient hominins must have taken on the path toward language. For example, active vocal interchange is widely recognized as foundational for vocal language, developing by the first three months of human life^29–32^; vocal interchange is also actively being evaluated in non-human primates^7,33^, although the results suggest considerably more restricted interactivity than in humans. Canonical babbling, developing usually by 7 months^34^, is recognized as a critical step in human vocal development since words in natural languages are overwhelmingly built from canonical syllables (e.g., baba and mama), and command over the use of canonical syllables has been proposed to have represented a critical step in human evolution, predating language^35–37^. Production of canonical syllables has never been reported to occur in non-human primates. Joint attention, often supported by vocalization and developing by 9–12 months^38,39^ is recognized as a critical foundation for vocabulary learning^40,41^ and is further seen as having been a necessary step in hominin evolution prior to the appearance of referential vocabulary^42,43^. Joint attention has been documented for apes trained by humans^44^ but has never been reported for non-human primates in the wild. Vocal imitation of well-formed speech-like sounds by very young infants is quite rare^45^, but becomes more common by the end of the first year^10^. Failures to demonstrate vocal imitation in non-human primates^46,47^, along with the obvious importance for word learning of the ability to imitate, has led to extensive speculations that vocal imitation capability must have been a key factor in human language evolution^36,47,48^.

All these developments (vocal interchange, canonical babbling, joint attention, and vocal imitation) correspond to stages in development of speech and ultimately vocal language in human infants^49^. And yet systematic protophone production precedes all of them, apparently beginning within the first days of life. We are pursuing research on the earliest protophones because the ability to produce vocalization flexibly appears to form a foundation without which none of the later stages considered above would be possible, a point argued extensively in prior publications from our laboratory^28,36,37,50^.

Protophones in the second half year, including canonical syllables, have long been recognized as precursors to speech^51–54^. But earlier protophones such as the primary vocal types of early infancy (vocants, squeals, and growls) that do not show well-formed consonant-vowel-like form, have largely been ignored in the discussion of language origins^55,56^, a discussion that has also failed to exploit the implications of findings indicating coordination of parent and infant vocalization in the first months of life^57,58^.

Granted, non-cry speech-precursor vocalizations have been reported in newborns^9^, and automated analysis of all-day recordings has suggested protophones may even occur in preterm infants in neonatal intensive care^59^. However, a closer look at vocalizations of preterm infants before their due dates is in order since the automated analysis that was used to assess vocal behavior of the preterm infants in the prior work of Caskey *et al*. was only modeled for older infants and suggested, at best, vastly lower rates of protophones than in full-term infants. Furthermore, the prior work was unable to assess occurrence in preterms in neonatal intensive care of the three primary types of protophones of the first month after full term birth^4^, and could not reliably compare protophone and cry rates.

It has been claimed that “cry is the primary means of communication for very young infants^60^ (p. 265)”. Moreover it has been thought that human vocalization *begins* with cry, and that speech-like vocalization emerges from the cry root^8^, with all newborn infant vocalizations being treated as some form of cry^6,61^. This expectation appears to be founded on the idea that humans begin with pan-primate vocal capabilities that only diverge from the primate pattern months after birth. Indeed, evidence suggests the “phee” call in the common marmoset may indeed develop *from* its cry^7^. Thus, it might be expected that prematurely-born human infants produce *only* cries before their due dates.

### Goals and rationale

Our intention is to evaluate the extent to which protophones, the earliest vocal precursors to speech, occur even in infants who are born prematurely and are still in neonatal intensive care, and in full-term infants starting in the first month after birth and continuing through the first year. Comparison of rates of protophone production with rates of cry can help put in perspective the origin of protophones, which have been thought by many to be based on cry. Yet protophones do not usually express distress (although they sometimes do^2^), and they are often produced with no obvious intention for anyone to hear them. Speculations about why they occur at all can best be made in the context of quantitative information about the extent and contexts of protophone usage. The comparison of protophone and cry rates may be expected to yield surprises, because prior research with infants beyond two months suggests protophones occur considerably more frequently than cries^2,3^.

Cries are often very salient when they occur, being usually both long and loud. This saliency may account for the traditional impression that cries are the predominant sounds of early infancy. Yet quantitative comparison of rates of occurrence is necessary in order to place the protophones in proper perspective and to form a better foundation for speculations about the importance of protophones in the origin of language.

In order to pursue this research, 40 all-day recordings were made using the LENA device^62,63^ from 20 preterm infants in neonatal intensive care 8 and 4 weeks prior to due date (-2 and -1 months of age). In addition we obtained all-day recordings from 9 full-term infants at 0 months and from 12 (including the 9) full-term infants at 1, 3, 6, 9, and 12 months, yielding 69 all-day recordings of full-terms. We randomly-selected 24 five-minute segments from each recording and conducted human coding to estimate cry and protophone volubility. A questionnaire completed by coders at the end of each five-minute segment indicated the extent to which there was infant-directed speech (IDS), adult-directed speech (ADS), and the extent to which the infant was alone or asleep. For preterms and full terms together, we thus examined human coding of >2600 five-minute segments, yielding >13,000 minutes of vocalization data.

## Results

**Figure 1:**
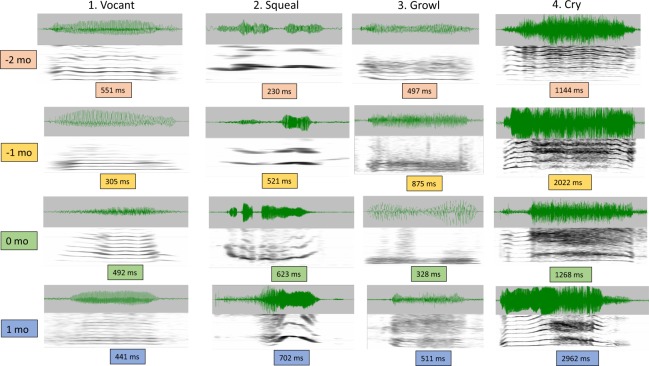
Spectrographic examples of vocalizations in preterm and full-term humans. Protophones (vocants, squeals, and growls) are displayed in the first three columns and cries in the fourth. Nearly all infants (all preterms and 11 of 12 full terms), produced all three protophone types (>40,000 observed). 18 of 20 preterms produced cries, whereas cries were represented for all the full term infants (>6,000 observed). A time domain display is provided at the top of each of the 16 examples, and spectrographic displays are provided at the bottom of each (range 4–5 kHz, 30 Hz bandwidth). The examples were produced by four different infants at each age in each of the four columns. Human infant sounds are extremely variable; every utterance of any of these infant vocal categories is distinct, yet the four categories of sounds represented in the figure’s columns correspond to auditorily identifiable exemplars of the categories as labeled. Wave files for each of the examples are available in Supplementary Information. (1) Vocants (or vowel-like sounds): By far the most frequent protophone category (>70% of all protophones at all ages), vocants show little interharmonic noise throughout all four exemplars. Vocants are usually produced in comfort. (2) Squeals: Produced with very high pitch, indicated by periods of widely-spaced harmonics; squeals represented ~7% of protophones, occurring in all but 3 infants. (3) Growls: these exemplars include considerable interharmonic noise and pitch lower than squeals. Some growls also include periods of very low pitch in a phonatory pattern called vocal fry (or pulse). Growls represented ~15% of protophones, and occurred in all the infants. (4) Cry: All four exemplars show human-typical, evenly-spaced harmonics (normal phonation) at the beginning of each of the cries, followed by significant and auditorily salient periods of dysphonation (sometimes called harsh phonation) in each of the four, corresponding to chaotic and/or subharmonic phonatory regimes. The pattern of normal phonation followed by dysphonation and then normal phonation again has been argued to constitute the most prototypical form of intense human infant cry^83^.

Contradicting the expectation that speech precursors emerge from cry, we found plentiful protophones even in awake −2 month-olds. Segments coded from the preterms included >11,000 protophones. The human coders indicated whether each protophone was a squeal, a vocant, or a growl (see Methods and Supplementary Information for details). Even for the youngest preterm infants (−2 mo), this coding yielded hundreds of exemplars in each of the three categories. Figure 1 provides spectrographic examples (wave files in Supplementary Information) of unambiguous cases for each category, selected to illustrate that preterm infants produced all three protophone types as well as cries that resembled the same kinds of sounds in full-term infants.

**Figure 2:**
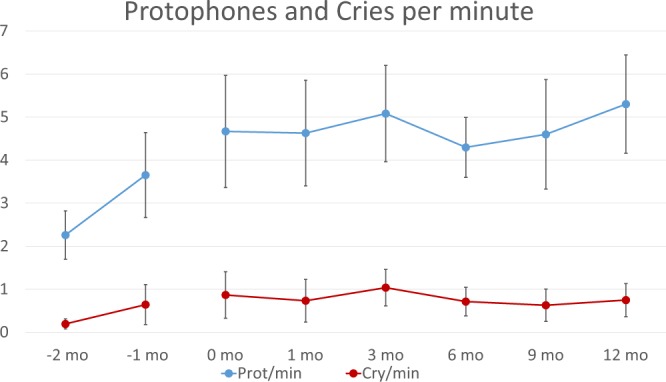
Vocalization rates in preterm and full-term humans. Protophones are infant sounds produced mostly in exploration or play rather than in social interaction. The figure illustrates that protophones/minute generally exceeded cries/minute by a factor of >5 (error bars represent 95% confidence intervals, computed based on the raw data from each infant and independent of the modeled data from the GEE analyses) in randomly-selected five-minute infant-awake segments from all-day audio recordings. Even in the preterm infants, protophones were plentiful, more than two per minute even at two months before due date, suggesting deep natural selection for the tendency to produce endogenous vocalization in humans. GEE analysis indicated a significant increase in the protophone rate for preterms from - 2 to -1 month of age. The differences between protophone and cry rates were massively significant by *t*-tests. The groups of infants recorded longitudinally were 20 preterms at -2 and -1 month of age (that is, 32 and 36 weeks gestational age), 9 full term infants at 0 months (that is, during the first month after full term birth) and the same 9 plus 3 additional full-term infants through 12 months. Each infant at each age provided an all-day recording from which 24 five-minute segments were randomly selected for the human coding that yielded the estimated rates of vocalization.

Figure 2 illustrates that, contrary to the common expectation, protophones in the randomly-selected segments occurred far *more* frequently than cries at both preterm ages (*p* < 0.0001 by *t*-test). For the full-term infants, the protophones also outnumbered cries dramatically, again at every age (*p* < 0.0001). Even the lowest rate found, 1.4 protophones/min for - 2 month-olds, corresponded to >80 protophones per waking hour. For both preterms and full terms, protophones outnumbered cries by a factor >5. Using Generalized Estimating Equations^64^ (see Methods and Supplementary Information for rationale), implemented in R, it was found that the full-term infants showed no significant effects of Age for either protophones or cries, but the preterms showed a significant effect for protophones (*p* < 0.0005) and a near significant effect for cries (*p* = 0.07), reflecting the increase in overall vocalization rate that presumably accompanied increasing respiratory sufficiency and maturational changes across age in the preterms.

**Figure 3:**
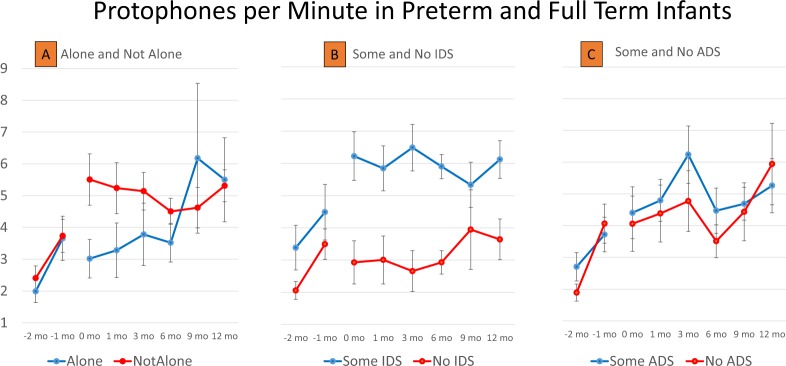
Protophone rates in preterm and full-term humans in differing circumstances. (**A**) Even when infants were alone (no one else was deemed present in the room by the coders), they produced large numbers of protophones (≥2/minute even in the -2 month old preterms), a strong indication of the endogenous nature of infant vocal exploration. The full-term infants, especially at younger ages, produced significantly more protophones when they were not alone, but adult speakers may have driven this effect by approaching very young full terms when they were producing protophones. Error bars are standard errors of the mean, computed based on the raw data from each infant and independent of the modeled data from the GEE analyses. (**B**) GEE analysis showed that full-term infants produced significantly more protophones when there was infant-directed speech (IDS), but once again parents may have driven the effect especially at the youngest ages, by engaging infants vocally when they heard the infants begin to vocalize. (**C**) Higher rates of adult-directed speech (ADS) were reliably related to higher protophone rates for the full-term infants, but the effect was not observed for all ages, and the effect size was much smaller than in the case of IDS.

Figure 3 provides illustrations of the copious occurrence of protophones regardless of circumstances. In these analyses, five-min segments were assigned to a high and a low condition in each case, splitting the data into two groupings with similar numbers of segments. Figure 3A shows that there were high protophone rates when awake infants were in a room with other persons but also when infants were alone, with an average of >3 protophones/minute in the alone circumstance (full terms: 220 alone segments, >4300 protophones; preterms 244 alone segments, >3100 protophones). The great majority of the protophones produced when infants were alone (full terms: 74%; preterms, 78%) came from five-minute segments with little or no crying (<5 cries/segment). GEE showed that full terms produced significantly fewer protophones when not alone (*p* < 0.05), a difference that was particularly obvious at younger ages. However, caregivers may tend to approach when infants are vocalizing, and thus caregivers may have driven the effect suggesting more vocalization when infants were not alone.

There has been considerable emphasis in speculation about the origin of language on parent-infant vocal interaction starting at 2–3 months^65^. Figure 3B shows that infants produced large numbers of protophones either with or without IDS. Still, full-term infants vocalized at nearly twice the rate during segments with infant-directed speech (IDS) than without it (GEE, *p* < 0.0001). Segments for preterm infants did not show significantly higher volubility with IDS, but parents visited their infants relatively infrequently in the hospital, and nurses presumably produced less IDS than their parents did^66^. It cannot be concluded with certainty that higher IDS *caused* higher infant volubility in young full term infants since the effect could have been influenced by parents’ choosing times to speak to infants when the infants were already engaged in a period of vocalizing.

The rate of IDS in the study was notable, suggesting a parental tendency to engage infants conversationally very early in life, even at home, a tendency that has been documented in recent research^67^. For full-term infants, caregivers used IDS in >65% of segments with infants awake. Even in the hospital, preterm infants heard IDS from caregivers and hospital staff in >33% of awake segments. While there appear to exist notable cultural differences in amount of IDS used by humans^68^, no quantitative study has indicated total absence of IDS in any culture. On the other hand our recent work found no caregiver vocalizations at all directed to three bonobo infants observed across the first year^5^.

Not just IDS, but also “overheard” speech may provide important input^69^, perhaps especially in cultures where parents speak to infants little in the first year. Infants produced high rates of protophones with and without ADS. Figure 3C illustrates that adult-directed speech (ADS) produced in the vicinity of full-term infants did correspond to higher protophone rates (GEE, *p* < 0.05), but the effect size was <1/3 as high as for IDS.

A final GEE analysis for full-terms was fit to the protophones with predictors Age, Alone, IDS, and ADS, where Alone, IDS, and ADS were treated as parameters with values from 1–5, corresponding to the questionnaire responses. A statistically significant effect was found for IDS (*p* < 0.0001); no interaction terms were significant. GEE analysis for the preterms yielded no significant main effects for protophones or cry on Alone, IDS, or ADS, but a single significant interaction of Age and ADS (*p* < 0.05) for protophones.

## Discussion

The roots of language appear to run deep in human infancy according to our data since as soon as infants were capable of vocalizing, even when born prematurely by more than two months, as soon as they could breathe independently, they began producing substantial numbers of protophones, the early precursors to speech. Full-term infants from the first month also produced protophones abundantly, and we observed no social circumstances under which protophone production was lower than 2.5 per minute for full-term infants. The *typical* rate for full-term infants was 4–5 per minute and not much lower for preterm infants at -1 month. Even at -2 months preterm infants produced more than 6 times more protophones/min than protophone-like sounds reported for three bonobo infants in the first year^5^.

We have reasoned that without the ability and inclination to produce such sounds, there would be only a much reduced basis for vocal interaction with infants, since parents tend to engage infants by responding to protophones and seeking to elicit them^36^. Considerable prior work has proposed that vocal interaction is fundamental in launching other developments required for language^58,70,71^.

It seems necessary to ask how the tendency of the human infant to produce protophones is sustained and to posit selection pressures that could have produced the ability and inclination to produce abundant sounds that express no obvious immediate needs. The proposal we have advocated in parallel with work of Locke is that such flexible, playful vocalization has long served to advertise fitness of the altricial (i.e., born helpless) human infant, who has a long path ahead of need for parental care^28,31,72–74^. As the reasoning goes, both modern and ancient hominins were altricial, presumably because bipedality had narrowed the human pelvis and thus required the fetal brain case to be smaller at birth in order to negotiate the passage—hence hominin infants *had* to be born altricial. As hominin evolution progressed, the mature head became progressively larger, requiring continuing limitations on the size of the infant head at birth and progressively longer periods of infancy and childhood. As hominin infants required longer periods of parental care, the selection pressure to advertise fitness also increased.

According to the reasoning, helpless infants can improve their prospects for survival and reproduction by convincing caregivers to invest extensively in them. Infant vocalization produced freely and comfortably can signal well-being, advertising the likelihood that investment in that particular infant is worth the effort. That vocalization can evolve as a fitness signal is well-documented across thousands of species of songbirds, hummingbirds, parrots, pinnipeds, cetaceans, and bats^20,75–82^. In these species, song-like vocalization advertises fitness to potential mates and to competitors. The selection principle is similar to that proposed for the human infant protophones; vocalization in circumstances that appear to involve little or no distress serve, in all these cases, to display well-being by e.g., advertising a healthy respiratory system, an ability to modulate the phonatory system, sometimes intricately, and by advertising the very fact that the organism is not in distress.

The current results suggest the selection pressure on fitness signaling has been sufficiently general to launch protophone production even when infants are born well ahead of schedule. The results further suggest that the roots of protophone production run so deep as to call into question the idea that speech-like vocalization is grounded in cry. Human language appears to have required special pressures to afford the production of sounds that are not bound to the expression of emotion, opening a path to a vastly more powerful communication system.

## Methods

### Approvals

The work described here was approved by the Institutional Review Boards of the University of Memphis, Memphis, TN and Women and Infants Hospital, Providence, RI. All methods were performed in accordance with the relevant guidelines and regulations of the IRBs. Informed consent was signed by parents of all the infants recorded.

### Participants and recordings

12 full-term infants of mid to high-mid SES were recruited in Memphis. All-day recordings of ~12 hours in duration were made throughout the first year. 20 low risk preterm infants without congenital abnormalities, born at 30-weeks gestational age or younger were recruited through Women and Infants Hospital, Brown University. These infants were in neonatal intensive care through 36 weeks gestational age. All the preterm infants had been extubated by 32 weeks and were able at least minimally to vocalize by that time. Seven of the mothers of the preterms were high school graduates and the remainder completed at least partial college. The preterm infants were recorded for 16 hours each, at both 32 and 36 weeks gestational age (-2 and -1 months of age respectively).

The recordings were made with the battery-powered LENA system^62^ worn in infant vests by full-term infants and placed in the isolette or open crib near the preterm infants’ heads, minimizing mouth-to-microphone distance in both cases. We selected 24 segments of five minutes each for human coding and analysis from each recording, at equal time intervals beginning with a semi-randomly-selected five-minute segment. A questionnaire item to determine if the infant had been asleep was administered to coders at the end of coding for each five-minute segment. Segments where the infant was asleep were excluded from analyses. >46,000 infant utterances were identified by the coders in these randomly-selected samples. Table S1 (Supplementary Information, SI) characterizes the sample, and the text of the SI elaborates on participants and recording procedures (sections S1.1.1-S1.1.3).

### Coding categories

Consistent with the goal of the research, the only categories of sounds quantified for the analyses were Protophones and Cries. Coders, however, classified utterances in more detail, using the categories: squeal, vocant, growl, whisper, ingress, wail, whimper, laugh, and other. Vegetative utterances such as burps, hiccoughs and sneezes were not coded. Squeals, vocants, and growls (which accounted for the vast majority of all assigned codes) were collapsed into the single category Protophone for analysis and the two distress types (wail and whimper, which accounted for the vast majority of the remaining codes) were collapsed to Cry for analysis. ~1% of coded utterances pertained to any category other than Protophone or Cry. The SI (sections S1.1.4-S.1.1.5) details the coding system and its rationale.

The distinction between the collapsed categories of Protophone and Cry is based on the fact that wails and whimpers include characteristics in both vocalization and facial affect that mark them as obligatorily negative—wails and whimpers are assumed to have been evolved as inherently negative emotional expressions. Sound alone is sufficient to identify both wails and whimpers reliably as distinct from other infant sounds, in particular from protophones^83^, which usually do not express negativity although they can do so on occasion^2,4^. Acoustically, wails consist of intense (loud) nuclei with variable phonatory regimes of substantial duration usually including substantial harsh quality^83^. Typical whimpers are less intense, have shorter nuclei, and are required by definition to include at least one glottal burst (see SI for illustrations). Wails can also include glottal bursts and/or catch breaths^83^.

The three primary protophones can be characterized acoustically^2,84^ as follows: vocants (often called vowel-like sounds) typically consist of nuclei with normal phonation at variable lengths and typically at moderate amplitudes; squeals include salient periods where pitch is typically double that of vocants, usually in loft (falsetto) register; and growls are usually lower in pitch than vocants, though growls are primarily characterized by a salient period of either harsh phonatory quality (manifest in subharmonic, biphonation, or chaotic vocal regimes) or pulse (vocal fry) register.

While the categories have these acoustic characterizations, coding is based first and foremost on intuitive judgments. In fact, the acoustic characterizations available in our own and related literature describe findings for auditorily-specified categories. The coding system is founded on the assumption that human caregivers have been naturally selected to recognize categories of infant sound because that recognition puts them at an advantage in nurturing their own offspring. Thus distress sounds must be identifiable without training, and by implication, other sounds must be differentiable from distress sounds. Laughter, similarly, must be identifiable and must be auditorily distinct from other sounds.

In accord with our reasoning, parents must be able to identify the protophones as the flexible product of infant vocal exploration, sounds that are not bound to any particular emotion (although of course each protophone type can accompany any emotion on particular occasions). The three principal subcategories of protophones appear to be the self-organized products of infant vocal exploration in the non-random space provided by the infant phonatory apparatus^28^. The explorations presumably yield certain categories preferentially, and infants tend to produce these favored categories repetitively. Following the same reasoning, it makes sense that parents must recognize the categories that infants favor.

The three primary protophone types appear to represent natural kinds^85^. They were reported spontaneously by parents as being “sounds the baby makes” in our first longitudinal research more than 40 years ago^86,87^. In ethologically-oriented coding of recordings from that first study of infants under 6 months of age, the same three types were proposed by coders. Subsequent research has supported the conclusion that indeed the vast majority of early infant protophones can be coded reliably and sensibly as having phonatory characteristics that pertain to one of these three protophones types.

### Coding procedure and questionnaire

Both Cries and Protophones were counted within this coding system as “breath groups”^88^, where each voiced period produced on a single egress was counted as one utterance. Coding was conducted in real-time. After coding each five-minute segment, coders responded to a questionnaire to determine for that segment (on a five-point scale) the extent to which 1) there was infant-directed speech, 2) there was other-directed speech, 3) the infant was alone in the room, and 4) the infant was asleep (see S1.1.7).

### Coders and training

Eighteen female master’s students in Speech-Language-Pathology served as coders. They were trained extensively as described in S1.1.8 before coding the recordings that were analyzed. Eleven individuals coded preterm recordings and eight coded full-terms (one coder worked on some recordings from both groups). Because coders worked in the project ten hours per week for a period of not less than two years and usually more, they could not be blinded in general to the research interests, and they were fully aware of whether they were coding full-terms or preterms. On the other hand, they were given no information about the particular infants they were assigned to, and their assignments with regard to infant age were ordered randomly. Still it was possible for them to discern much about the infants and families while they were listening to the recorded segments.

The primary task of coding training is not to teach coders to recognize the categories used in the coding system (distinguishing wails and whimpers from protophones is a natural human capacity), so much as to teach them to systematically use the labels the coding system assigns to those natural categories and to teach them to count utterances using the breath-group criterion.

### Coder agreement

As reported in S1.1.9, there were several types of coder agreement studies conducted both within the largely disjunct coder groups (preterm and full-term) and between them. In all cases the agreement as measured by correlations of numbers of Protophones counted exceeded 0.8, and in most cases the same was true of Cry counts. More important, however, was the fact that coder differences on counts as indicated by coefficients of variation (COVs) showed that the massive differences between Protophone and Cry rates illustrated in Fig. 2 were more than 6 times larger than estimated coder differences.

### Statistical procedures

The analyses regarding protophone and cry usage (Fig. 2) were conducted with paired comparisons *t*-tests for each age independently. Generalized Estimating Equations (GEE)^64^ were used for additional analyses. GEE is a modeling approach that can account for fixed and random effects but is preferable to more traditional mixed-models frameworks for semi-longitudinal research, especially when there are correlations among data from participants across conditions, and when the number of observations varies for participants within or across conditions. In essence the approach offers an assessment that estimates on a principled basis the means and standard deviations relevant for the analysis while taking into account intragroup correlations and variations in numbers of observations. The GEE method requires an assumption of a link function between means and a linear predictor (we chose a linear link) and the specification of a covariance structure (we chose an exchangeable covariance structure). Other advantages of GEE over traditional mixed models are that it requires no normality assumption, and it will result in consistent estimates of means even if the correlation structure is misspecified.

The GEE analyses were conducted on the preterm samples separately from the full-term samples, because the groups of participants were disjunct in the two cases, and because of the fundamental differences in circumstances of recording (in the hospital vs at home). The statistical approaches are discussed in more detail in the SI (section S1.1.10).

## Supplementary information

Supplementary Information

Cry, 0 months

Growl, 0 months

Squeal, 0 months

Vocant, 0 months

Cry, 1 month

Growl, 1 month

Squeal, 1 month

Vocant, 1 month

Cry, -1 month

Growl, -1 month

Squeal, -1 month

Vocant, -1 month

Cry, -2 months

Growl, -2 months

Squeal, -2 months

Vocant, -2 months

Figure S1

Figure S2

Figure S3

Figure S4

Figure S5

Figure S6

Figure S7

## Data Availability

The authors will supply the spreadsheets from which the data for this article were computed on request and these will also be deposited in the Open Science Framework. These spreadsheet data will allow readers to recompute the values or analyze the data in ways that differ from those reported here. In addition the R code for the GEE analyses will be deposited in the Open Science Framework.
