# Supplementary material for: Preterm and full term infant vocalization and the origin of language
Source: Sci Rep. 2019 Oct 14;9:14734. doi: 10.1038/s41598-019-51352-0 (PMC6791864; doi:10.1038/s41598-019-51352-0)
Supplement: Supplementary file 1 — Supplementary Information [file 41598_2019_51352_MOESM1_ESM.docx]

**Supplementary Information for**

**Preterm and full term infant vocalization**

**and the origin of language**

**D. Kimbrough Oller*^1,2,3^, Melinda Caskey^4^, Hyunjoo Yoo^5^**

**Edina R. Bene^1^, Yuna Jhang^6^, Chia-Cheng Lee^7^, Dale D. Bowman^1^,**

**Helen L. Long^1^, Eugene H. Buder^1, 2^, Betty Vohr^8^**

^1^University of Memphis, Memphis, Tennessee, USA

^2^Institute for Intelligent Systems, University of Memphis, Memphis, Tennessee, USA

^3^Konrad Lorenz Institute for Evolution and Cognition Research, Klosterneuburg, Austria

^4^Kaiser Permanente, Oregon, USA

^5^University of Alabama, Tuscaloosa, Alabama, USA

^6^Chung Shan Medical University, Taichung, Taiwan

^7^Portland State University, Portland, Oregon, USA

^8^Alpert Medical School of Brown University, Women and Infants Hospital, Providence, RI, USA

*Correspondence to D. Kimbrough Oller, University of Memphis, 4055 N. Park Loop, Rm 4023, Memphis, TN 38152. [koller@memphis.edu](mailto:koller@memphis.edu)

**Table of contents**

**S1: Methods details**

***S1.1: Methods details regarding all-day audio recordings of human preterm and full-term infants***

**S1.1.1 Participants**

**S1.1.2 Device and recording procedure**

**S1.1.3 Recordings and dataset, including Table S1**

**S1.1.4 Coding categories**

**S1.1.5 Acoustic display Figures S1-S7 illustrating vocalizations of preterm and full-term infants**

**S1.1.6 Coding procedure and questionnaire**

**S1.1.7 Coders and training**

**S1.1.8 Coding protocol**

**S1.1.9 Coder agreement**

**S1.1.10 Statistical treatment**

**S2: Expanded reasoning about the results**

***S2.1 Other thoughts: Robustness of spontaneous vocalization and comparison of human coding results with LENA automated analysis results***

***S2.2 Other thoughts: Caregiver responsivity and nurturance***

***S2.3 Final thoughts: The evolution of language***

**S3: Audio examples**

**From Figure 1, main text**

Fig1: 0m_Cry.WAV

Fig1: 0m_Growl.WAV

Fig1: 0m_Squeal.WAV

Fig1: 0m_Vocant.WAV

Fig1: 1m_Cry.WAV

Fig1: 1m_Growl.WAV

Fig1: 1m_Squeal.WAV

Fig1: 1m_Vocant.WAV

Fig1: minus1m_Cry.WAV

Fig1: minus1m_Growl.WAV

Fig1: minus1m_Squeal.WAV

Fig1: minus1m_Vocant.WAV

Fig1: minus2m_Cry.WAV

Fig1: minus2m_Growl.WAV

Fig1: minus2m_Squeal.WAV

Fig1: minus2m_Vocant.WAV

**From section S1.1.5**

FigS1_WailCry_Preterm_Minus1Month.wav

FigS2_Vocant_Preterm_Minus1Month.wav

FigS3_WailCry_Preterm_Minus1Month.wav

FigS4_Vocant_Fullterm_0months.wav

FigS5_WailCryWithCatchBreath_Fullterm_0months.wav

FigS6_Whimper_Fullterm_0months.wav

FigS7_Whimper_Preterm_Minus2Months.wav

**References**

**S1: Methods details**

***S1.1: Methods details regarding all-day audio recordings of human preterm and full-term infants***

**S1.1.1 Participants in the all-day recordings**

The 12 full-term participants for the present research came from a longitudinal study of vocal development focusing on typically developing infants. All these infants were recruited by advertising and/or word-of-mouth, in most cases prior to birth, and were enrolled after consent being granted by parents to the University of Memphis IRB approved protocol. The infants came from families where maternal education included some college in four cases, a BA degree in five cases, a master’s degree in two cases, and a PhD in one. All were white.

The Memphis longitudinal study involves many components, but for the present purposes, the relevant part is the all-day recordings that were scheduled to occur for all the infants, starting optimally in the first month after birth. The study of these full-term infants was planned to include all-day recordings at every month starting at 0 months. For 3 of the 12, it was not possible to schedule a recording at 0 months, so there were 9 infants represented at 0 months in the research reported here. All 12 infants were represented in the coding for all-day recordings at 1, 3, 6, 9, and 12 months. Brief summarial data about this study on full term infants have been presented previously for comparison with non-human vocalization observations[^1^](#_ENREF_1), but without age-specific data, nor was any of the data on the preterm infants discussed or involved.

The 20 preterm infants were recorded in a different effort at Women and Infants Hospital, under a similar IRB-approved protocol. These infants were born at 30-weeks gestational age or younger (with a mean = 27.2 weeks; mean birthweight 904 grams), and received care in a Neonatal Intensive Care Unit (NICU) through at least 36 weeks. All were low risk and relatively stable preterms, without congenital abnormalities, blindness, SNHL or cerebral palsy. For 10 of the preterm infants, the NICU was open-bay with up to 12-20 infants in a bay so that there was considerable activity nearby, intermittently throughout the day, often with nurses, physicians, other hospital personnel and parents present[^2^](#_ENREF_2). For the other 10 preterm infants the NICU was private room, with less activity and noise. Possible differences between vocal rates of the preterm infants in the open-bays and private rooms as well as possible differences in the amounts of vocalization they heard are being investigated for a separate article. Parents were encouraged to visit, and consequently during the recordings, voices of both hospital staff and family of the infants could often be heard.

The preterm infants selected for the study had achieved medical and respiratory stability and all had been extubated by 32 weeks. Therefore they were at least minimally able to vocalize by that time, although their level of maturation and respiratory capabilities clearly placed some limits on the quality of the sounds produced. Each of the 20 infants was recorded all-day for 16 hours at both 32- and 36-weeks gestational age while still in the hospital. Sixteen of the infants were White, 1 was Black, and 3 were Hispanic. Seven of the mothers were high school graduates and the remainder completed at least partial college. Days on oxygen for the preterm infants ranged from none to 120 (mean = 35.7 days) and days in the NICU ranged from 46-134 (mean = 87.5). No infants were discharged on oxygen.

**S1.1.2 Device and recording procedure for the all-day audio recordings**

The battery-powered LENA recorders[^3^](#_ENREF_3) are about the size of an iPod, and for full-term infants, fit in the chest pocket of special clothing so that mouth-to-microphone distance is nominally 5-10 cm. The system can record up to 16-hours. The sampling rate is 16 kHz, yielding recordings that are quite satisfactory for the human coding and analyses of the article. Automated analysis of the LENA recordings is obtainable, but the present work relies instead on human coding, which is more reliable although more time consuming by far.

For the full-term infants, the recording procedure was standard for the LENA system[^4^](#_ENREF_4). Briefly, parents were provided with the appropriate clothing, were given instructions regarding how to charge the recorder overnight, then how to turn it on and place it in the child’s clothing at wake up time, then how to turn it off at bedtime and return it to the staff of the research project for processing. At bath times, parents were instructed to place the recorder near the bath area, as close to the infant as possible without letting it get wet. The recordings lasted 12 hours in most cases.

For the preterm infants, the procedure had to be adapted of course because of the size of the infants. The recorders were turned on by a research assistant after charging and were placed in the isolette or open crib either in a small vest fitted specifically for the infant or immediately adjacent to the infant’s head. Because not all the preterm infants could wear the special clothing, the recorder distance was more variable (~7-15 cm) than in the case of the full-term infants.

**S1.1.3 Recordings and dataset for the human all-day audio recordings, including Table S1**

A single all-day recording (16 hours for each recording for preterms infants and an average of 12-13 hours for each recording for infants at all of the full-term ages, 0-12 months) was obtained. The LENA software automatically breaks the recording down into consecutive 5-minute segments that can be conveniently accessed. We selected 24 such segments for human coding and analysis from each recording, at equal time intervals beginning with a semi-randomly-selected (RS) 5-minute segment within the first 1/24^th^ of the all-day recording, and then 23 additional segments were selected at equal time intervals so that the entire day was representatively sampled.

Table S1 provides a characterization of the sample. The preterm component was entirely longitudinal with a recording for each infant at both ages: at 32 weeks gestational age (-2 months, that is 2 months before the due date) and at 36 weeks (-1 month). The full-term component was also longitudinal, but three of the infants had no recording at 0 months.

Altogether there were 109 all-day recordings, from which (24x109) 2616 five-minute segments were human coded. To afford the possibility of optimally comparing vocalization rates across age, we took a measure of sleep through a questionnaire responded to by coders at the end of coding each 5-minute segment (Was the infant asleep? Scale 1-5). As expected, infants were deemed asleep during segments at a notably diminishing rate across age (ranging from 65% of segments for the -2 month-olds to 47% of the time for -1 month olds and <30% of segments for full terms). 47,888 infant utterances (protophones+cries) were identified by the coders in these RS segments when the infants were awake. Table 1 indicates the number of RS 5-minute segments where the infant was deemed by the coder to be awake the whole segment and the number protophones found in each such segment.

**S1.1.4 Coding categories**

The coding focused on the presumed precursors to speech, the protophones[^5^](#_ENREF_5), but also provided counts for cries, which were treated as the other most prominent kind of sound produced by infants in the first half year. Laughs were coded as well, but they were quite infrequent, essentially absent before 6 months, accounting for ~ 1% of utterances at 6 months, and only 8% at 9 and 12 months. Consequently, our analysis left laughs out of the picture altogether, to be the subject of a subsequent study. Further the three protophone types that were coded (squeals, growls and vowel-like sounds) were collapsed together to simplify an already complex set of analyses.

The reason the distinction between cry and protophones is crucial for this work is that cry maintains an obligatory strong connection with the expression of distress across early infancy and includes both acoustic and facial characteristics marking it as cry. Protophones, on the other hand, do not show an obligatory connection with any particular affect condition at any age. In fact all the protophones we have examined (squeals, growls, vowel-like sounds, raspberries, voluntary ingressive sounds, frication sounds, and both marginal and canonical babbles) have been reported by parents and/or shown in our coding data to be used on different occasions with positive, neutral, *and* negative affect[^6^](#_ENREF_6)^,^[^7^](#_ENREF_7).

|  |  | **Months of age** | | | | | | | |
| --- | --- | --- | --- | --- | --- | --- | --- | --- | --- |
| **Participant** | **Sex** | **-2** | **-1** | **0** | **1** | **3** | **6** | **9** | **12** |
| PT1 | F | 8/27 | 19/144 |  |  |  |  |  |  |
| PT2 | F | 16/118 | 23/391 |  |  |  |  |  |  |
| PT3 | F | 3/120 | 2/262 |  |  |  |  |  |  |
| PT4 | F | 7/56 | 16/113 |  |  |  |  |  |  |
| PT5 | M | 23/254 | 19/686 |  |  |  |  |  |  |
| PT6 | F | 20/610 | 18/885 |  |  |  |  |  |  |
| PT7 | F | 15/641 | 14/506 |  |  |  |  |  |  |
| PT8 | F | 19/384 | 11/386 |  |  |  |  |  |  |
| PT9 | M | 8/273 | 22/1038 |  |  |  |  |  |  |
| PT10 | F | 2/217 | 15/931 |  |  |  |  |  |  |
| PT11 | M | 6/304 | 5/146 |  |  |  |  |  |  |
| PT12 | F | 5/299 | 10/513 |  |  |  |  |  |  |
| PT13 | F | 4/160 | 11/758 |  |  |  |  |  |  |
| PT14 | F | 7/163 | 8/438 |  |  |  |  |  |  |
| PT15 | F | 4/118 | 17/385 |  |  |  |  |  |  |
| PT16 | F | 10/228 | 17/980 |  |  |  |  |  |  |
| PT17 | F | 6/239 | 8/845 |  |  |  |  |  |  |
| PT18 | F | 2/74 | 7/254 |  |  |  |  |  |  |
| PT19 | F | 3/24 | 12/170 |  |  |  |  |  |  |
| PT20 | F | 2/22 | 2/88 |  |  |  |  |  |  |
| FT1 | F |  |  | 20/756 | 22/920 | 15/471 | 20/271 | 10/379 | 13/206 |
| FT2 | M |  |  | 19/650 | 19/506 | 10/455 | 17/363 | 19/937 | 19/804 |
| FT3 | M |  |  |  | 20/513 | 19/605 | 21/483 | 19/445 | 21/1035 |
| FT4 | M |  |  | 18/253 | 19/476 | 17/544 | 22/589 | 22/553 | 19/464 |
| FT5 | F |  |  |  | 16/131 | 10/207 | 16/488 | 12/186 | 20/361 |
| FT6 | F |  |  | 22/462 | 19/533 | 19/492 | 19/453 | 21/573 | 20/493 |
| FT7 | M |  |  | 15/437 | 16/644 | 12/339 | 11/251 | 9/102 | 8/247 |
| FT8 | M |  |  | 19/491 | 12/181 | 11/337 | 16/381 | 11/265 | 21/418 |
| FT9 | F |  |  |  | 24/482 | 24/454 | 24/476 | 24/331 | 24/653 |
| FT10 | M |  |  | 10/226 | 19/341 | 11/131 | 14/117 | 16/273 | 11/285 |
| FT11 | F |  |  | 19/381 | 21/474 | 17/266 | 12/327 | 18/211 | 13/241 |
| FT12 | F |  |  | 16/85 | 22/140 | 19/231 | 14/233 | 21/406 | 17/358 |

**Table S1.** We evaluated all-day recordings from each preterm (PT1-PT20) and full-term infant (FT1-12), semi-randomly selecting 24 five-minute segments (see text). The leftmost number in each cell indicates the number of semi-randomly-selected five-min *segments* where the infant was awake the whole five minutes, and the rightmost number indicates the number of protophones found in the semi-randomly-selected segments with the infant awake.

The protophones are spontaneous in the sense that no particular emotional condition or external stimulus is needed to inspire their production, and in this way they appear to provide the groundwork for the development of the speech capacity. A recent study from our laboratory[^1^](#_ENREF_1) addressed the possibility that an analogous distinction between cry and protophones might exist in one of our closest phylogenetic relatives, the bonobo, *Pan paniscus*. Protophone-like sounds did seem to occur in the three captive infants (always with their mothers) that were studied, although they were used with more restricted functions and at far lower rates than in the human infants. Infant bonobo screams were deemed analogous to human crying, although we observed no sounds that were similar to prototypical human crying from an acoustic perspective. Consequently, it appears human infants show signs of selection pressure on both the capacity and the inclination to produce protophones, and thus, differentiating protophones from cry, and maintaining counts of both in our research, is considered critical.

Coders were encouraged to use intuition in differentiating cry from protophones (the task involved a forced choice), since we operate on the assumption that human listeners have a natural capacity for drawing the distinction[^7^](#_ENREF_7)^,^[^8^](#_ENREF_8). Indeed, as will be seen below, coder agreement on the distinction is good, although some utterances mix features of cry and protophones, yielding intermediate cases. Yet if it were not possible regularly to draw the distinction between cry and non-cry, caregivers would be handicapped in assessing infant needs—it seems clear that selection pressure throughout hominin history must have been applied to making cry a signal of distress and to leaving the protophones free to vary in emotional expression. But simply to define cry as a distress signal is insufficient to differentiate it from any of the three protophone types, because these have all been shown to manifest positive, neutral, and negative *facial* affect on different occasions of usage, all three affect types occurring with each protophone type by not later than 3 months[^7^](#_ENREF_7), when positive affect is first systematically manifest in smiling. Furthermore, negative *vocal* affect is also discernible at good reliability in all the protophones on some occasions of usage[^9^](#_ENREF_9).

We use two operational criteria intended to help coders deal in a relatively consistent way with ambiguous cases in categorizing cry vs. protophones. The first criterion concerns the part of any voiced utterance called the “nucleus”, analogous to the “vowel” in a spoken syllable, the part that includes relatively continuous periodic or semi-periodic voicing on a single egressive breath. Consider a comparison of two such nuclei of ~1 sec with no consonant-like portion, no glottal or supraglottal bursts, sharp ingressive catch breaths, or glottal holds, and assume the two nuclei have similar durations and amplitude contours. Protophone exemplars of this sort sometimes reveal strong negativity in audio alone but still can be consistently distinguished auditorily from cry that meets all the same criteria. The precise acoustic basis for this distinction is under study, but for the present purposes, suffice it to say that such negative protophones are often called “yells”, “angry yells”, or “whines”, while the cries maintain a special character, involving a combination of acoustic characteristics described in a recent article[^10^](#_ENREF_10), and they give the auditory impression of being more involuntary (compelled by intense distress) than the otherwise similar protophones.

The second criterion for differentiating cry and protophones includes two parts, based on very brief acoustic events, the cry glottal burst, sometimes referred to as a “cough”[^11^](#_ENREF_11)^,^[^12^](#_ENREF_12) and the cry catch breath. The glottal burst is egressive, lasting 20-40 ms, and occurring either just before or just after a cry nucleus, being " ...accompanied by spasmodic movement of the abdominal wall and forceful expulsion of air (p. 124)"[^13^](#_ENREF_13). The catch breath is ingressive, occurring just after an intense cry nucleus often long enough to expel the essential entirety of the infant’s vital capacity. While glottal bursts often occur in intense cry with long and high-amplitude nuclei, glottal bursts can also occur in vocal “fussing” or “whimpering” at much lower amplitude and with much shorter nuclei (often < 100 ms). This kind of whimpering, with at least one glottal burst and a nucleus was included in the cry category for the coding system used for the present research, although the perceived negativity of whimpers is usually deemed lower than in other cries. On the other hand, glottal bursts all by themselves (with no voiced nucleus immediately following or preceding them) are deemed “other” within our coding system, that is, neither cry nor protophones. Similarly a catch breath is a marker of cry in our system, as long as the ingress appears to occur either because the infant is seemingly out of breath after a long cry nucleus or a long sequence of whimpers on a single egress and thus is obliged immediately to inhale, or because the cry nucleus is sufficiently intense that the catch breath is deemed auditorily to be a natural product of that intensity. A nucleus that might otherwise be deemed ambiguous (as cry or protophone), may well be heard as cry if it is followed immediately by such a catch breath. Protophones, in accord with our coding instructions, involve no such catch breaths (although of course inhalation does take place between protophones, and sometimes infants play with ingressive-egressive sequences where the ingresses are voiced) and no glottal bursts.

Both cries and protophones are counted within this system in accord with a “breath group” criterion[^14^](#_ENREF_14), where each voiced period produced on a single egress is counted as one utterance. Also each ingress or each cessation of voicing that is long enough for an ingress (whether one is heard or not) is deemed an utterance boundary, although a glottal hold followed by further voicing without and intervening ingress, either in cry or protophones, no matter how long, does not terminate an utterance.

Cry and protophone utterances are thus counted in an analogous way in accord with the breath-group criterion, which breaks cry bouts into individual cries of roughly similar dimensions to protophones produced in bouts. The criteria for the cry/protophone distinction designate topographical features of cry (glottal burst, catch breath, specialized nucleus) that allow relatively straightforward judgments in most cases and that invoke specialized vocal cry markers of distress, as opposed to other presumable vocal markers of distress that do not include the cry topography. By including utterances that are called in the common parlance “fussing” within the cry category, the proportion of counted cries increases but still falls far below counted protophones.

**S1.1.5 Acoustic display Figures S1-S7 illustrating vocalizations of preterm and full-term infants**

Supplementary Figures S1-S7 show spectrographic examples of cries and protophones for both preterm and full term infants, indicating features differentiating cries and protophones—these figures supplement those in the main text, Figure 1. The figures include differentiated nuclei, along with glottal bursts and catch breaths, which occur only with cry and whimper. Also the supplementary Figures illustrate typical features of utterances from the preterm infants.

**
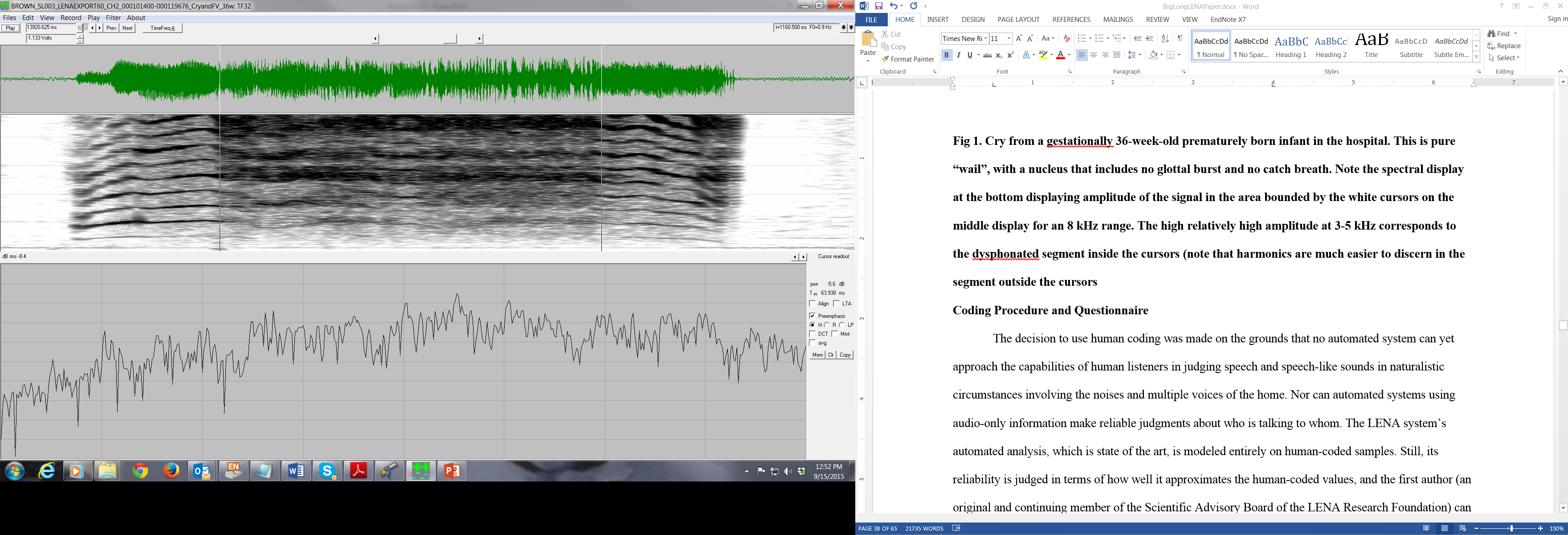
**

Fig. S1. An example cry displayed in TF32[^15^](#_ENREF_15) from our recordings of a prematurely born infant at 36 weeks gestational age still in the hospital. This is pure “wail”, perceived as very intense cry, with a nucleus that includes no glottal burst and no catch breath. Figures S1-S7 include at the top, a waveform, in the middle a spectrogram with time on the x axis, frequency on the y axis, and amplitude presented as darkness of the trace, and at the bottom a time slice spectral display with amplitude on the y axis and frequency on the x axis. The time slice spectral display pertains to the first 40 ms of the period bounded by the two vertical cursors that run from top to bottom of the middle display (the remainder of the period bounded by the cursors shows a similar spectral tilt pattern). The equally spaced vertical lines in the time-slice spectral display indicate 1 kHz intervals. The relatively high amplitude at 3-5 kHz corresponds to the dysphonated segment inside the cursors in the middle display (note that harmonics are much easier to discern in the segments outside the cursors). This pattern with strong dysphonation and high amplitude in the mid (nominally 3-5 kHz) range is typical of intense cry and absent in vocants with normal phonation (vowel-like sounds). Especially notable in intense wail cry is that the “spectral tilt” results in emphasis on the middle frequency range such that amplitude in the 1500 Hz is routinely very low compared to the amplitude in the middle range. The opposite is true in vocants.

**
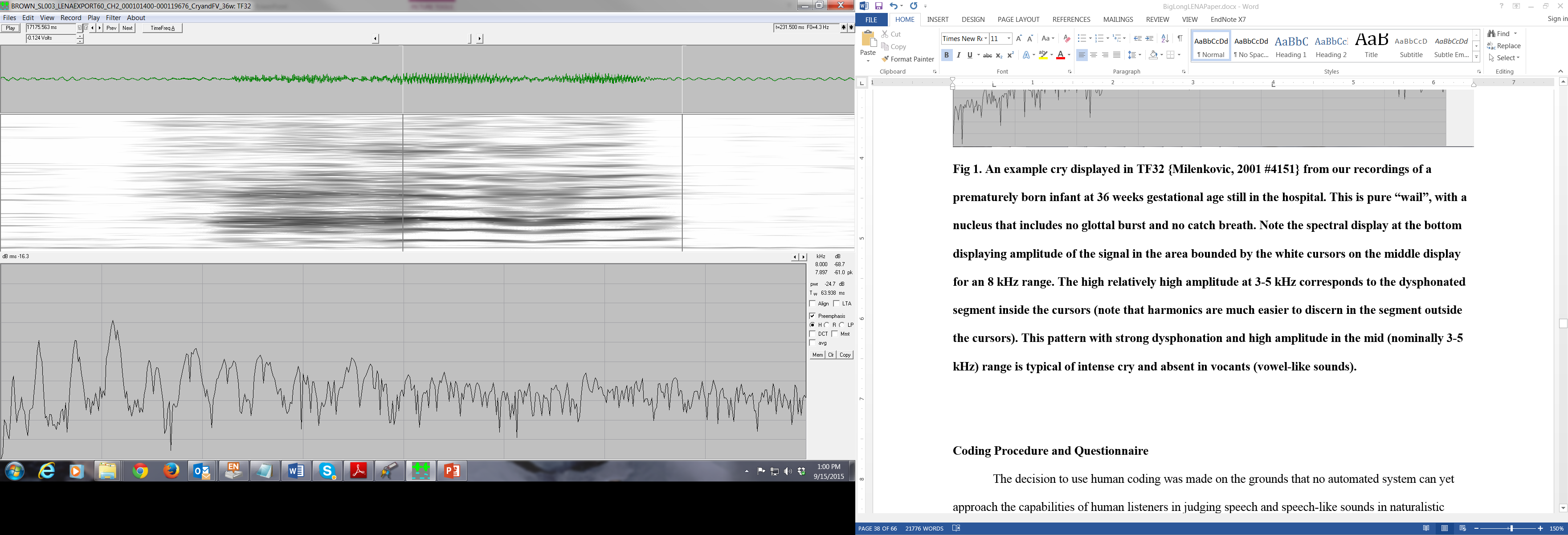
**

Fig. S2. A vocant from the same infant as in Fig. S1 during the same recording at 36-weeks. Note much lower amplitude overall, but also higher relative amplitude in the low frequency range from <1500 Hz, a spectral tilt pattern typical of normal phonation in vocants. The blue arrow marks the point of maximum amplitude in the period up to 40 ms after the left vertical cursor, and as in Fig. S1, a similar spectral pattern occurs through the period between the vertical cursors. Even the much noisier portion of the signal preceding the cursors in the middle display (a corresponding spectral curve is not displayed) does not involve the pattern of high relative amplitude in the 3-5 kHz range as seen in the cry segment of Fig. S1. In the middle display, harmonics are discernible some of the time as equally spaced horizontal lines (for example beneath the red arrow on the right), a pattern seen in normal phonation. Note that the pattern of phonation is unstable, with harmonics being erratically discernible (red arrow on the left pointing to a period of little discernibility of harmonics, which suggests dysphonation, while the arrow on the right shows high discernibility of harmonics). Erratic discernibility of harmonics corresponding to intermittent dysphonation was common for vocants produced by the preterm infants.


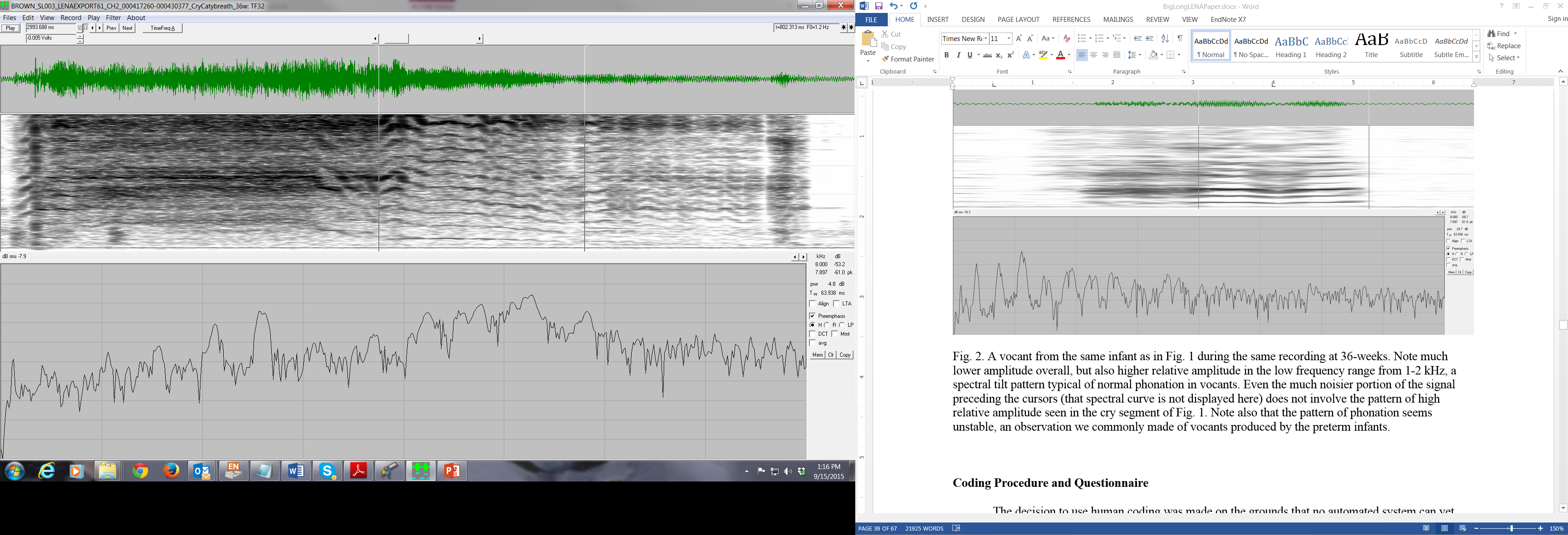


Fig. S3. A cry beginning with a long and complex wail and ending with a catch breath (a rapid inhalation) at the very end, the noisy segment bounded by red arrows. This cry comes from an infant at 32 weeks gestational age and shows again the characteristic cry pattern of low spectral tilt for the period of time bounded by the cursors in the middle display (the period of maximal discernibility of harmonic energies), with maximum amplitude above 5 kHz (light blue arrow). The cry also includes considerable dysphonation, little discernibility of harmonics, and low spectral tilt during most of the first half of the utterance, all features characteristic of wail cry.


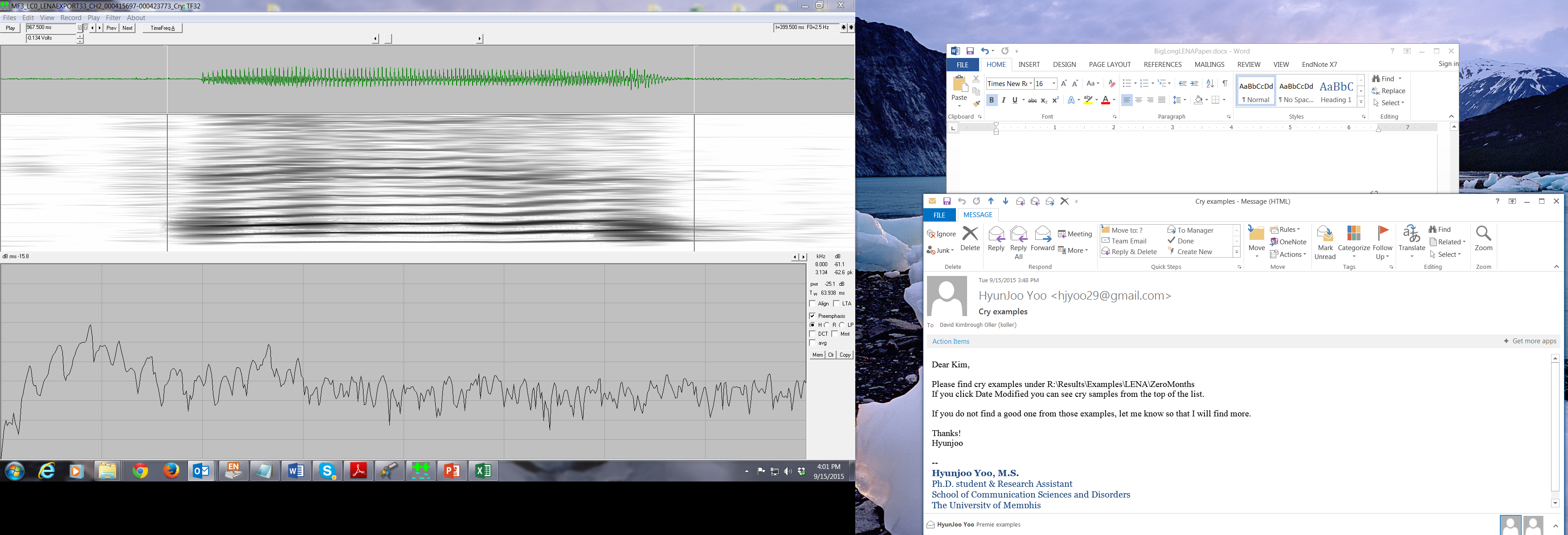


Fig. S4. This is a vocant from one of the recordings of a full-term infant at 0 months. Note the smooth normal phonation with harmonics discernible throughout the utterance (contrasting with Fig. S2, where harmonics not discernible throughout the infant vocant), and the fact that the spectrum shows tilt toward low frequencies, with a peak under 1 kHz (light blue arrow), the typical spectral pattern for vocants.


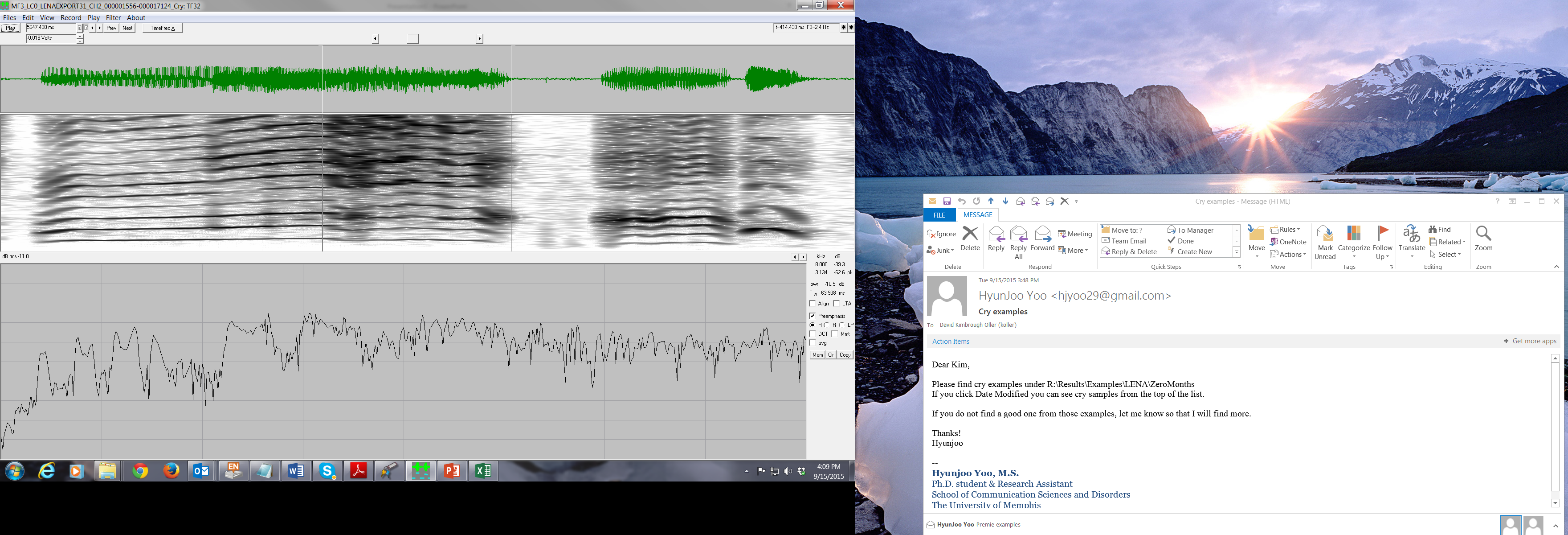


Fig. S5. A cry from a recording from the same 0 month-old in Fig. S4. The utterance includes a portion bounded by the white cursors that includes the low tilt characteristic with highest amplitude between 3 and 5 kHz. A break in the phonatory pattern (without inhalation) occurs in the second half (dark blue arrow), then phonation continues, followed by a sharp catch breath (with widely spaced harmonics corresponding to the very high perceived pitch), bounded by red arrows.

**
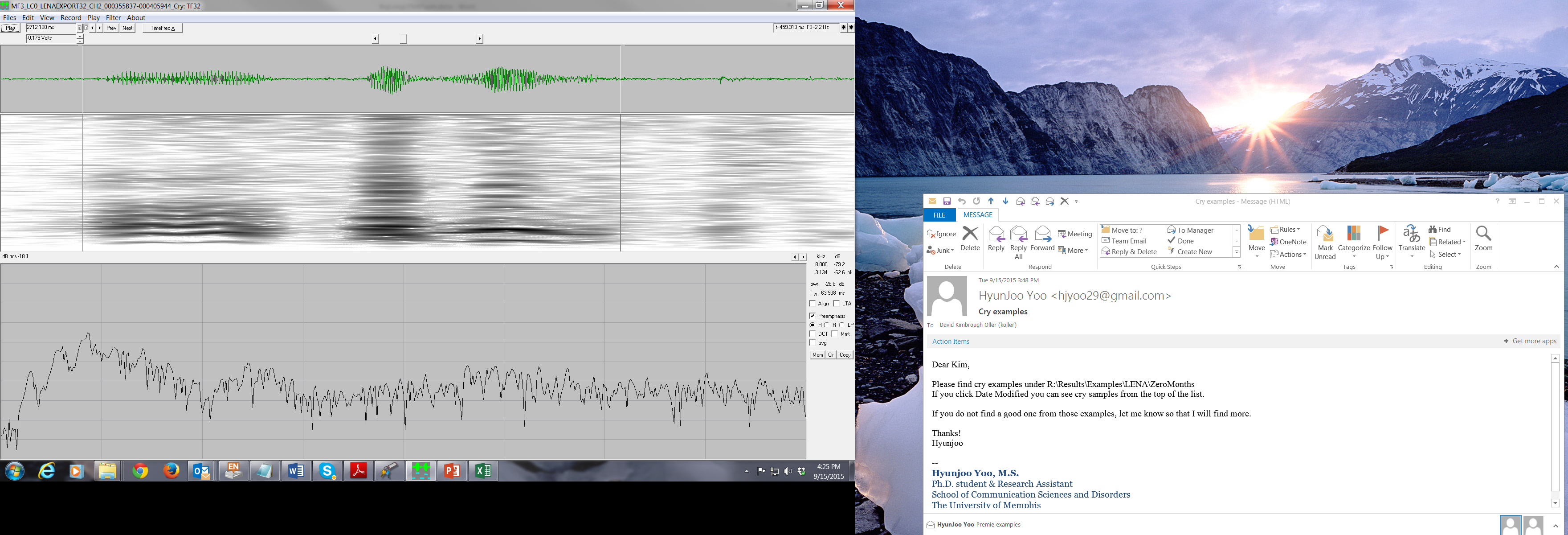
**

Fig. S6. This is a whimper from the same 0-month-old infant as in Figs. S4 and S5. The yellow arrow marks the longer nuclear portion of this whimper (we call it a “whine” onset to the whimper), and note that during this relatively low amplitude period (yellow arrow) of the whole utterance, the spectral display does not indicate the pattern of low spectral tilt usually found in cry, even though the whine is also interpreted as transmitting negative affect. The light blue arrow on the spectrum marks the peak frequency of energy, just below 1 kHz, a tilt pattern typical of vocants. The dark blue arrow marks the glottal burst (which might be identified as a cough, if isolated) while the red arrow marks the shorter nuclear portion of the whimper, following the glottal burst (a pattern typical of glottal bursts in whimper).

Fig. S7. This is a whimper from a -2 month-old preterm infant. It consists of two units in a single breath group, each unit displaying the most typical pattern for whimper in that each begins with a whimper burst (dark blue arrow) and ends with a whimper nucleus (red arrow). One reason for providing this particular example is that it shows clearly that the very preterm infants sometimes did produce whimper, even though they did so far less frequently than full-term infants. Crying was present in the preterms much more frequently than whimper.

**S1.1.6 Coding procedure and questionnaire**

The decision to use human coding was made on the grounds that no automated system can yet approach the capabilities of human listeners in judging speech and speech-like sounds in naturalistic circumstances involving the noises and multiple voices of the home. Nor can automated systems using audio-only information make reliable judgments about who is talking to whom. The LENA system’s automated analysis, which is state of the art, is modeled entirely on human-coded samples. Still, its reliability is judged in terms of how well it approximates the human-coded values, and the first author (an original and continuing member of the Scientific Advisory Board of the LENA Foundation) can attest to the fact that everyone associated with development of the system acknowledges human coding as the gold standard. The goal of the present work is to obtain maximally reliable estimates of infant volubility. Consequently we used human coders for the work.

In order to make it possible to acquire a large human-coded dataset, we implemented a real-time coding method. The data collection at all ages of the present study involves listening to each assigned 5-minute segment in real-time and pressing the appropriate key in the AACT computer-based coding environment for each of the infant protophones (vocants, squeals, growls) plus each occurrence of cries (wails), whimpers, and laughs. Wails and whimpers were collapsed to a single “cry” category in the analyses. An “other” category was also allowed, but like laugh, it was rarely used, and was not included in the analyses.

After the 5-minute listening and coding period for each segment, the coders responded to a number of questions; the key ones were as follows. 1) Did any other person talk to the baby? This could be the parent or another adult or child. 2) Did any other person talk to someone else? For example, two adults might have talked to each other, or a person might have talked on the phone. 3) Do you think the baby was alone in the room? And 4) do you think the baby was asleep? The questions were answered on a 5-point scale, where 1 indicated never, 2 some of the time, 3 about half the time, 4 most of the time, and 5 the entire time.

**S1.1.7 Coders and training**

The coding team consisted of 18 normally-hearing female Masters students in Speech-Language-Pathology from the University of Memphis’ School of Communication Sciences and Disorders. All had been trained in phonetic transcription during their program of study. Training for the coding of infant vocalizations began with a two-hour introduction to infant vocalizations with both audio and audio-video examples presented primarily by the first author, whose work for decades has been focused on infant vocal development and the establishment of appropriate descriptive schemes for prelinguistic sounds[^7^](#_ENREF_7)^,^[^16-18^](#_ENREF_16). Also assisting in the training during the first phase, which involved preparation to code the data were the third, fifth, sixth, and eighth authors, all PhD students at the time working on infant vocal development under the direction of the first and ninth authors, and the fourth author, the Project Coordinator. All the training personnel had had considerable experience by that time in coding infant vocalizations.

The coding team was also introduced in this initial session to AACT (Action Analysis Coding and Training[^19^](#_ENREF_19)), the software coding environment utilized and largely developed in the Memphis laboratories in collaboration with Intelligent Hearing Systems of Miami, FL. The system, under development since the 1980’s, affords numerous conveniences for coding in both real-time and repeat-listening, and participants learned what they needed to learn to use the AACT system well enough to begin coding their training samples within an hour or so. Thereafter, a series of practice tests were given during which each coder worked independently or with a senior coding partner (a PhD student or another member of the coding team who had gone through the training in a previous year), coding infant vocal samples much like the ones that would be coded when the real data collection began. Several such rounds of coding and reviews of the results with the training staff occurred during a period of 6 to 8 weeks. These reviews consisted of at least one meeting per week of the group as a whole and individual meetings occasionally of the first or fourth authors with individual coders whose results seemed to warrant special attention and/or correction.

In order for a coder to be deemed ready to begin coding for data collection in the study, it was required that their final rounds of coding fall within 10% of the protophone and cry counts in a gold standard set of five sessions where the original coding had been done by the first author. Also the coders were required to have diverged by an average of not more than one on the scale from one to five on the questionnaire items pertaining to IDS, ADS, Alone, and Sleep. A check on the agreement levels for the coders was made at the end of this period of training for each (to be reported below) based on ~84 five-minute segments from four different infants that were not among the 100 infants in the study.

There were four training waves based on students entering the coding team at various points across Spring 2016 through Fall 2018, all conducted as described above, with the coding team always consisting of at least 10 members, new ones entering and others graduating each year. Generally coders worked on the coding team for at least four semesters and one summer before graduating.

There was special attention paid to training for coding of the preterm infants, because no one prior to our own work has done such coding to our knowledge. In fact about half the data on the preterm infants had been coded in our laboratory during 2013-2014. These data will be referred to below to compare for agreement with the more recent round of coding, which we deem more reliable because it was based on updated and more rigorous training. The coders who received this special training for the current study (mostly in Fall of 2018) worked with examples from the actual data set (6 five-minute segments selected on the basis of the earlier coding in 2013-2014) for training purposes. The primary focus of this training was on recognizing cry and protophones in infants whose respiratory systems were weak and thus produced sounds that differed often notably from those of more mature infants. We sought to ensure that the coders could apply the differentiation of cry and protophones in a way that was as analogous as possible to that used at the older ages. Again the gold standard coding was done by the first author, and all the coders had to meet standards similar to those described above in order to be deemed certified to participate in coding the preterm data.

**S1.1.8 Coding protocol**

There were eight coders who worked on the full-term data and 11 on the preterms. There was one coder who participated in both groups (thus there were 18 coders in the entire effort). Each infant in both the preterm and full-term groups was assigned to a specific coder; coders were assigned to multiple infants, but the protocol specified that they work through the entire data set for each infant before proceeding with the next infant to which they were assigned. Furthermore coding of each recording was completed before another recording was coded, and the 24 five-minute segments were coded in their chronological order during the recording day. The order of coding for recordings within infant was, however, random to prevent the coders from knowing the age of the infants directly.

The reason for yoking of coders to infants is that one learns how to account for the context of infant utterances while listening to recognize speakers, family patterns, and vocalization patterns within infant. This experience helps make the coder more confident and reduces the stress of the coding. We presume coding is more accurate when coders are yoked to infants. Furthermore, coder biases complicate within-infant interpretation (for example for changes across age) if there are multiple coders for a single recording or infant; thus all 24 five-minute segments from an individual infant were coded by the same individual, and all recordings available for that infant were coded by the same individual. The five-minute coding period for each segment was followed by about one minute for the questionnaire that followed and for saving of files.

**S2.1.9 Coder agreement**

We have made several assessments of coder agreement relevant to the current study. Because the coders for the preterms and full terms were largely disjunct groups, we will report here relevant agreement data both within the two coder groups and across them.

The primary issue at stake in assessing agreement is its relation to the key findings of the study, in particular the finding that preterm as well as full-term infants produced large numbers of protophones, and further that the number of protophones was greater than the number of cries by five fold or more in both infant groups. To assess the likelihood that individual coder bias characteristics (e.g., one coder tending to code more utterances overall than another coder or tending to code more utterances as protophones rather than cries than another coder) might have exaggerated the findings or might have yielded an unreliable outcome (i.e., a new set of coders would not produce data replicating the pattern of results), we determined the agreement levels on numbers of protophones and cries coded across pairs of coders who independently coded an identical set of segments in several circumstances. The agreement statistics of most interest concern the discrepancy between the results of any pair of coders on the number of cries and the number of protophones determined through the coding of each, as well as the correlation of segment-by-segment results for the two coders.

All 18 coders coded (almost all of) 84 five-minute segments independently. These represented 21 randomly-selected segments from each of four all-day recordings, all from different infants, none of whom were among the 20 preterms or 12 full terms in the study. These 84 segments were a post-training set designed to document agreement, that all coders working in our laboratories for the past four years must complete as a final step in the training process.

To make a balanced comparison across the coders of the preterms and the coders of the full terms, we paired the 7 full-term coders who had *not* coded preterms with 7 coders who *had* coded preterms, leaving out of the comparison the coders who had done the least coding in the main study. This cross-group comparison yielded strong evidence that coding biases or other effects could *not* have played any significant role in the main findings of the study (namely the results indicating that preterms and full terms produced very large numbers of protophones, and that both groups showed much larger numbers of protophones than cries).

*Cross-group comparisons*: First, both the 7 individuals from the full-term coding group and the 7 individuals from the preterm coding group found large numbers of protophones (3.2/min and 3.0/min) in the ~84 segments that were coded by both groups independently (a number that is lower than the estimates in Figure 2 of the main text, because the agreement sample included sleep segments). The similarity of the numbers of protophones found by both coder groups suggests little if any biasing of the groups with respect to each other. Second, the correlations between numbers of vocalizations coded by the 7 individuals from the full-term coding group and 7 individuals from the preterm coding group was 0.86 and 0.85 for protophones and cries respectively (n ~ 84 for each of the 49 pairings of coders). Third, the mean proportion of protophones (number of protophones / (number of cries + number of protophones)) was 0.94 for the full-term coders (15 times more protophones than cries) and 0.93 for the preterm coders (14 times more protophones), illustrating that a massive difference between cry and protophone rates was seen by the coders from both groups. Third the coefficient of variation (COV) for protophone counts in the full-term coding group (standard deviation of the number of protophones across the seven coders divided by the mean number of protophones for the seven coders) was 0.12 and in the preterm coding group (for 11 coders), 0.25. For cry counts, the COVs were considerably higher, 0.58 for the full-term group and 0.51 for the preterm group. However, these relatively high COVs do not threaten the interpretation of the results in the main text because they are very small with respect to the effect sizes reported there (see next paragraph). Still, we are working on improving coding agreement regarding cry counts. Our speculation about the coding discrepancies is that in intense cry bouts, many breath groups occur in quick succession, and real-time coding may not provide the optimal method for acquiring counts based on the breath-group criterion for such intense cry bouts.

To place these COVs in the perspective of the effect sizes reported in the main text, consider the differences found between the numbers of protophones and cries produced by infants in both the full-term and preterm infants, as represented in Figure 2; across ages in both groups of infants, the difference is greater than 500% (often much greater); by comparison a maximum estimated difference between coders across groups based on COVs is about 83% (the sum of the larger of the two COVs for protophones [the preterm coder COV, 25%], and the larger of the two COVs for cries [the full-term coder COV, 58%]). Hence, the difference as reported in Figure 2 is more than 6 times larger than the mean cross-group coder difference as estimated from the COVs.

Another key point is that all the coders coded large numbers of protophones at all the ages in the main study. Even the preterm infants showed high numbers of protophones. Based on the higher of the two COVs for protophones, the numbers of protophones found for infants across the ages might be expected to vary by as much as 25% if a new group of coders had been assigned to the study. Yet even the lowest mean protophone value reported in Figure 2 of the main text for any age (2.26/minute in the -2 month olds) would still, even if reduced by 25%, correspond to >800 protophones per 8-hour day. All the coders thus found large numbers of protophones, even in the preterm infants in neonatal intensive care, and this finding is not threatened by coder differences.

*Coder bias evaluation:* Agreement between coders can also be assessed in terms of bias of each individual, as represented for example by the tendency of individuals to code fewer or more items within each category. Here we offer statistics on the number of protophones and cries coded by each of the 14 coders (the 7 preterm and 7 full-term coders) on the agreement samples discussed in the previous section. For the ~ 84 five-minute segments that were coded by each of the 14 individuals for that study, the mean number of protophones was 2.84/min with a standard deviation of 0.56/min, reflecting a COV of only 20%, again a very small difference compared with the >500% difference between protophone and cry rates in Figure 2. For cries, the mean across the 14 coders was 0.20/min, and the standard deviation was 0.10/min, with a COV of 52%, again a very small value with respect to the >500% difference between protophone and cry rates in Figure 2. Another way to look at the minor effect of coder bias in the context of the present study is to consider the proportion of items coded as protophones across the 14 coders. The mean proportion was 0.93 with a standard deviation of 0.04. The lowest proportion of protophones for any of the 14 coders was 0.85, indicating clearly that all of the coders found massively more protophones than cries in the agreement sample and that coder bias was most likely a very small factor in our results, extremely unlikely to have affected our main conclusions.

*Within-group comparisons*: Both the 7 preterm coders and the 7 full-term coders that were tested for across-group agreement were also subjected to within-group comparisons, where every possible pairing of 7 within each group (*n*=21 in both cases) was tested. The results for agreement were also supportive of the idea that coder differences could not have invalidated the main study findings. The mean correlations between numbers of protophones and numbers of cries across the 21 pairings of coders from the full-term coding group was 0.86 and 0.81 (*n* ~ 84 for each of the 21 pairings) respectively; for the preterm coding group, the protophone and cry correlations were 0.83 and 0.81.

*Cross-generation coding comparison*. Half the recordings from the preterm infants had been coded previously by a 2013-2014 team. The five coders of these recordings had been trained under an earlier protocol (we presume the newer one is better designed). The correlation of protophone counts for segments coded by the five earlier coders and the 11 coders of the current preterm study was 0.88, and for cries the correlation was 0.87, based on 480 segments (24 semi-randomly selected from each of 10 infants, not excluding infants asleep). For both coder groups the protophones outnumbered cries dramatically, by 8-fold in the earlier group and by 17-fold in the latter. The more recent group coded about 85% more vocalizations overall, a difference that we think is attributable to changes in the training protocol (from 2013 to 2016 and later) that focused more attention in recent time on the breath-group issue, a change that presumably resulted in the more recent coders (all those in the current study) designating a larger number of utterances because they learned through the training to attend more intensely to breath-group breaks in clusters of utterances occurring in real-time. Recognizing these breaks is not easy to do in real-time, and we are confident, based on reviews of coded sequences during training, that the more recent coders had learned to perform the task more reliably and had produced more veridical values.

*Full-term coder agreement study*: A final agreement analysis was conducted by randomly assigning “agreement” coders of the full-term coding group to recode segments that had previously been coded by a primary coder (namely another member of the full-term coding group). All the coders in the full-term group received such agreement coding assignments, which were semi-randomly made, ensuring that every agreement coder was paired with every other coder for some segments and ensuring that segments from an individual infant’s recordings would be coded by the agreement coder in clusters, the way segments had been ordered for the primary coders. Also, both primary and agreement coders finished an infant’s data before going on to another infant’s data, although they were both blinded about how the assignments had been made. Also every coder received assignments for recordings from a variety of ages.

212 segments were assigned for this study. The results showed a 0.89 correlation on protophone counts and a 0.68 correlation on cry counts. The relatively low correlation on cry counts appears to have been attributable mostly to a handful of segments at the one-month age that were coded very discrepantly. Also for any individual infant and recording there were never more than 6 segments assigned for agreement coding, which may have limited the power of the correlational analysis. Still, the overall picture was very much as in the other agreement assessments: both the primary and agreement coders showed very large discrepancies between the number of protophones and the number of cries, with the primary coders indicating 8 times more protophones than cries in the segments assigned, and the agreement coders indicating 10 times more protophones than cries. And both groups showed very large numbers of protophones, 3.5-3.6 protophones per minute, a value somewhat lower than full-term infant values in Figure 2 of the main text, presumably because the agreement segments were selected semi-randomly and thus included some segments where infants were asleep.

This agreement study was also used to assess the questionnaire results, which were based on scaled judgments ranging from 1 to 5. The key questionnaire items resulted in correlation across the primary coders and the agreement coders of 0.59 for IDS, 0.69 for ADS, 0.46 for Alone, and 0.76 for Asleep. These correlations should be interpreted in light of the fact that the average differences between judgments on any question were small—0.37 for IDS, 0.55 for ADS , 1.10 for Alone, and 0.50 for Asleep.

Perhaps a more meaningful analysis of agreement on the questionnaire items is based on evaluating the coder agreement for the splits that were actually used in the analyses presented in Figure 3 of the main text. IDS and ADS were split so that “some” IDS or ADS (a questionnaire response of >1) was contrasted with “no” IDS or ADS (a questionnaire response of 1), while Alone and Asleep were split so that Alone or Asleep “the whole time” (a questionnaire response of 5) was contrasted with “not Alone or Asleep the whole time” during the segment (a questionnaire response of <5). These splits yielded relatively similar numbers of segments in the two groups in each case. Using these splits as the basis for the agreement data, the two coders had 71, 79, 59 and 76% agreement on segments designated as “some” IDS or ADS and Alone or Asleep “the whole time”. The correlations for the split data were 0.72, 0.77, 0.61, and 0.81 for the four questions respectively.

The research reported here provides indications that all three of the primary protophone types occurred in both full-term *and* preterm infants. The examples in Figure 1 of the main text are intended to illustrate that there exist unambiguous cases of each protophone type, especially in the preterms for whom no such demonstration has previously been published. However, we have withheld performing statistical tests comparing rates of usage of the three protophone types based on the current real-time coding. The coder agreement based on the cross-generation coder study of real-time counts for two of the three subcategories of protophones was notably lower than for protophones or cries. The correlations across the two coder groups were 0.43 for squeals and 0.44 for growls (0.87 for vocants). While these values are highly significant statistically (*p*<.0001), we have resolved to hold off on statistical tests for the protophone subcategory usage until we complete repeat-listening coding (which takes at least five times as long as real-time coding), which we presume will yield higher correlations among coders.

**S1.1.10 Statistical treatment**

The analyses regarding protophone and cry usage (Figure 2, main text) were conducted with paired comparisons *t*-tests for each age independently. More elaborate procedures were not deemed necessary because the trends were unambiguous and coder agreement results (see above) showed that there was essentially no possibility that the differences between protophones and cries had been significantly influenced by coder biases or other differences among them.

Analyses on the effects of Aloneness, IDS, and ADS (Figures 3A-3C) were based on Generalized Estimating Equations (GEE)[^20^](#_ENREF_20) because the effects were more diverse and subtle. GEE is an advanced form of modeling where fixed and random effects can be accounted for, but where there are additional advantages over traditional mixed models approaches. The GEE analyses were conducted on the preterm samples separately from the full-term samples, because the groups of participants were disjunct in the two cases, and because of the fundamental differences in circumstances of recording (in the hospital vs at home). GEE is preferable over traditional mixed models frameworks for semi-longitudinal research when there are correlations among data from participants across conditions, and when the number of observations varies for participants within or across conditions. In essence the approach offers an assessment that estimates, on a principled basis, the means and standard deviations relevant for the analysis while taking into account intragroup correlations and variations in numbers of observations. The GEE approach also has the advantage of requiring no normality assumption.

Figures S8A-S8C provide the “predicted” or modeled means and standard errors based on the GEE analysis of the data shown in Figure 3A-3C of the main text. Both the means and the standard errors are based on the GEE model and differ from the standard error values in Figure 3 of the main text. The GEE approach is motivated by the fact that all the data can be sensibly utilized to model a best estimate of the real population of events based on the sampled data, an estimate that in principle should improve upon the estimate that can be based on a presentation of raw data as in Figure 3 of the main text. The GEE graphs thus facilitate our interpretations. The main effect of Aloneness and of ADS in the full-term infants can be interpreted based on the graphs as being moderated across age to a substantially greater extent than the effect of IDS. Further the IDS effect is clearly greater in size than that of either Aloneness (by a factor of ~2) or ADS (by a factor of >3).

Figure S8: Compare these GEE predicted results with those of Figure 3 in the main text. As can be seen the GEE predicted values appear “smoothed” across ages within conditions as opposed to the raw means and standard errors of the means for the data displayed in the main text. The standard errors, represented here as vertical bars at each age, are based on the GEE model and are not identical to those of the raw data as represented in Figure 3 of the main text.

**S2: Expanded reasoning about the results**

**S2.1: Other thoughts: Robustness of spontaneous vocalization and comparison of human coding results with LENA automated analysis results**

This work highlights the robust presence of protophones even in infants two months premature and still in the hospital; they produced >2 protophones per minute in RS samples when they were awake. At one-month premature, the value increased by more than 60% for the same infants. Protophone volubility was higher in the full-term infants, ranging from 4.35-5.30 per minute across the 6 ages, but the substantial occurrence of protophones in the preterm infants suggests that the “vocal playground” that has long been recognized to occur in 3-6 month-old full-term infants[^16^](#_ENREF_16)^,^[^21^](#_ENREF_21) is already operative for infants at 32 weeks gestational age (-2 months). Projecting the amount of vocalization for a day, based on the RS sampling and the length of the recordings, the values reported here suggest that infants two months premature would have produced many hundreds of protophones/day, and older infants through 12 months, perhaps 3500 per day. These numbers suggest considerable vocal activity that we presume forms a critical basis for vocal communication learning.

The volubility level reported here is the first evaluation based on human coding of protophone and cry production on young preterm infants still in the hospital. Caskey et al.[^2^](#_ENREF_2) provided prior estimates based on the LENA automated system for data based in part on the same recordings. The current work offers a way to directly compare the volubility estimates for the two methods, but the outcomes of the comparisons vary based especially on age. Still, at all ages, the mean automated estimates for protophone production across infants or groups were *substantially lower* than the human coding estimates, in part due to design of the automated method, which is constructed to be conservative in assigning speaker labels, and thus to maximize the likelihood that an item identified as the child’s voice is indeed the child’s voice. In particular, the automated system is designed to label cases where two voices (or a voice and some other noise) occur simultaneously as “overlap”. Human coders are often able to reliably recognize the infant voice during overlap, and this may be the primary reason the counts of human coders are much higher than the automated ones.

In the data here, the human coded values can be estimated to have been ~13 times higher in the preterm infants than the automated values and ~2.3 times higher than the automated estimates in the full-terms. The much greater discrepancy between the estimates in preterms and full-terms suggests that the automated method is much more accurate for full-terms, which is predictable, since the modeling upon which the automated system is based utilized human coding samples from full-terms only (no preterm data were available at the time the modeling was done).

Of similar importance is the demonstration from the present work that infant protophone production (assessed in terms of frequency of occurrence of utterances, that is, breath groups), is the overwhelming mode of vocalization in the human infant, at every age evaluated. Informal inquiry with audiences at conferences on child development, as well as our longitudinal diary-based research on caregiver opinion regarding rate of occurrence of various vocal types in infants, suggests that cry is generally believed to be considerably *more* frequent than protophones in young infants, and the opinion of a variety of authors in print seem to support that expectation as well[^12^](#_ENREF_12)^,^[^22^](#_ENREF_22)^,^[^23^](#_ENREF_23). The facts as revealed in the current work show that protophones occurred several times more often than cry across the range of ages in the study for randomly-selected samples in the home or in the NICU.

Our coding was done in real-time, and data were not obtained in that coding for duration or amplitude of cry and protophones. We suspected (and acoustic comparisons in full-term infants confirmed) that when cry did occur, it tended to be longer and louder than protophones, which presumably makes cry particularly salient and highlights the need for parental reaction—subsequent work confirms that presumption[^10^](#_ENREF_10). Protophones, in contrast, usually present information about infant well-being rather than immediate need, and can thus often (perhaps usually) be taken as playful activity, giving reassurance to the caregiver and requiring no immediate action. Because of its high salience, we reason then that cry may be estimated by parents and others to occur more frequently than it actually does in comparison to the less emotionally salient protophones.

***S2.2 Other thoughts: Caregiver responsivity and nurturance***

It has been suggested that spontaneous vocalization in the human infant has been under selection pressure in hominin history, presumably because it signals fitness of human infants, whose altriciality makes them particularly needy with regard to caregiver investment[^18^](#_ENREF_18)^,^[^24^](#_ENREF_24). Assuming this is true, hominin caregivers have also presumably long been under selection pressure to recognize the vocal signaling of infants through protophones (human infant cry has also been posited to constitute a fitness signal in addition to being a signal of need[^25^](#_ENREF_25)). Furthermore, the tendency of human caregivers to actively engage infants in vocal interaction suggests they *seek to elicit protophones* as fitness signals. In any case, caregiver-infant vocal interaction has long been posited to form a crucial environmental niche for vocal communication development[^26-28^](#_ENREF_26).

The present data offer notable new perspectives on caregiver-infant vocal interaction because the data are based on all-day recordings that were sampled randomly to maximize the representativeness of the vocal patterns, and because human coding allowed concurrent assessment of caregiver and other adult vocal activities in the presence of the infants, as well as assessment of infant wakefulness. The data suggest especially that full-term infants produce more protophones when caregivers talk to them, that is, when the caregivers produce infant-directed speech (IDS) than when they don’t.

The higher rate of infant vocalization in IDS did not apply to the preterm infants. Two possible reasons come to mind. First, the preterm infants were in the hospital and consequently were in the presence of their parents only during designated hospital visiting periods, which could not have been well-coordinated with periods of infant wakefulness, whereas full-term infants were at home where their parents could interact with them more freely. Nurses appear to have interacted vocally with the infants much less than parents. These differences in circumstances of the full-term and preterm infants could have accounted for the lack of a clear IDS relation with volubility in the preterm infants.

Second, the difference in IDS effects across the preterm and full-term infants could be a result of the nature of *possible* interaction in the two cases. The preterm infants faced a greater survival challenge, their vision was considerably less developed, their breath control was considerably less advanced, and both their physical strength and wakefulness were more limited. Consequently, even if parents wished to interact vocally with the preterm infants, it may have been difficult to do so. Parents presumably recognized the more limited vocal and facial interactive capacities of the preterm infants and produced a different pattern of interaction. This speculation is supported by the fact that the volubility of infants at -1 months (after they had matured considerably) was 60% higher than at -2 months. Thus, we reason, the higher infant volubility during IDS in the full-term infants was presumably partly the product of parent recognition that their infants born at term were capable of interaction, that is, able to vocalize, maintain eye contact, and remain awake.

Further it is possible that the preterm infants were simply not maturationally ready for vocal interaction. Perhaps one of the products of natural selection on vocal tendencies in the human infant has been to ensure that the infant can begin to interact vocally promptly after (full-term) birth, but not necessarily early in gestation.

However, it is not clear from the present data, how soon infants were truly interacting vocally. Prior research on parent-infant vocal interaction has tended to focus on 2-4 months[^29-31^](#_ENREF_29), and in general it has been concluded that by that age, infants have indeed begun to interact actively, that is to say, to show signs of stimulability, reacting with higher rates of vocalization when parent vocalizations are contingent on infant vocalizations. Similarly, the literature suggests a growing tendency from two months forward for infants to take turns in vocalization rather than clashing with parent utterances[^32^](#_ENREF_32)^,^[^33^](#_ENREF_33).

Notably, no prior research to our knowledge has reported higher infant volubility during IDS than in its absence, nor has it reported higher protophone volubility with parent stimulation in 0-1 month olds. Prior research has almost always been based on short samples usually from laboratory recordings. Our data are among the first to examine randomly-sampled day-long recordings for such purposes. In this context we found higher infant volubility during IDS at 0 and 1 months, and are compelled to consider how this could be so if it is true that infants are not yet capable of systematic adjustment of vocal rate in response to caregiver attention by 0 and 1 months? Our speculation is that the effect at this early age may not be driven by infant responsivity, but by the caregiver’s ability and tendency to engage the infant vocally when the infant is awake and primed to vocalize. According to our reasoning, the mother approaches and stays with the infant, vocalizing interactively in part because she realizes the infant can, by this age, maintain some visual contact, yielding the impression to her that her vocalizations are drawing the infant into interaction[^8^](#_ENREF_8), and the appearance of interaction may also be supported by unconscious coupled oscillation. The mother, according to this reasoning, has chosen an intelligent course, because the very activity she engages in appears to provide the infant with a learning niche that may foster the growth of the infant’s interactivity. As the infant matures and learns, true infant vocal responsivity begins to play a role in interaction, according to our reasoning. And of course, coupled oscillation may play a role throughout the learning process. The present data do not directly support this speculation, but they are consistent with it. Future research on the timing of vocalizations during such randomly-selected samples from day-long recordings may be able to more fully evaluate the relative contributions of mother and infant to vocal interactions across the range of ages considered here (and see Hilbrink et al.[^34^](#_ENREF_34), for a recent view).

Apparent discrepant results on infant abilities and interaction patterns between prior research and the current work may be due to the fact that prior research has overwhelmingly focused on selected periods where infants have been brought into the laboratory for study during periods designed by experimenters, and where only optimal data (with high volubility and low negativity) within that context have been considered. Everything else has usually been thrown out. Random sampling from all-day recordings puts us in the proverbial whole new ball game. A typical feature of prior infant vocalization research based on short-term sampling was to focus on periods where the infant was particularly vocal, on the assumption that this focus would produce a representative picture of the infant’s maximal capabilities. Yet that focus may also yield very different outcomes with regard to responsivity of the infant than if the focus is shifted to the whole infant day (see Oller et al.[^1^](#_ENREF_1) for pertinent data). We presume the naturalistic whole day is the more important point of observation.

Of additional importance is the role the present results may play in laying groundwork for prediction of speech and language disorders. The results provide preliminary estimates of human infant volubility across the first year, but the sample is relatively small, and estimates of variance across infants based on the sample are presumably insufficient to provide trustworthy clinical guidelines regarding variations from normal volubility that may signal risk of disorder. The data on relations between IDS and infant volubility and on volubility when infants are alone and awake are definitely suggestive of the importance of determining whether variations from a normal pattern of such effects is predictive of later disorders.

Notably, the present work offers important additions to the prior work of Caskey et al.[^2^](#_ENREF_2)^,^[^35^](#_ENREF_35) using all-day recordings. That work provided important perspectives on the language environment of infants born prematurely and still in the hospital, but the present work has provided new evidence on particular circumstances of interaction, and new estimates of the frequency of IDS in the hospital. Perhaps even more importantly, the present work offers more reliable estimates of rates of vocalization, both protophones and cry in preterm and full-term infants, estimates that will need to be refined for future clinical applications through study of larger numbers of infants at each age, and through work on predictiveness of early individual differences in vocal patterns with regard to later development of language and cognition.

***S2.3 Final thoughts: The evolution of language***

Understanding the origins of human language, we think, requires inquiry into both the deep roots of vocal communication in human infants and evaluations of animal communication. Yet current language evolution research focuses not on the foundations of communication in a variety of species, foundations that can presumably provide a view of the distant past in animal communication, but overwhelmingly on speculated changes in the hominin line itself at the level of advanced features of language such as semanticity (including the use of symbolic lexical items, that is, words) or syntax (the ability to combine words productively in systematic ways to form sentences that produce meanings that are not the sum of the lexical parts)[^36-41^](#_ENREF_36). Since such features provide little basis for direct comparisons with other species in the wild (other species do not have such capabilities, although human-reared animals have been shown able to learn some such features to a notable extent[^42^](#_ENREF_42)), modeling of possible evolution of such features is not well-grounded in biology.

Our contention is that seeking the ancient roots of language requires seeking the roots of animal communication through comparative research (and we grant that many, especially non-linguists, agree with us). We believe the search is likely to be most fruitful (and here we depart from many comparative researchers) if we focus on communication in various primate species at the very beginning of life, where commonalities between humans and our nearest relatives are most likely to be discernible. We highlight also the somewhat obvious point that there is need for quantification of infant vocal characteristics within and across species to provide perspective on the extent to which the foundational capabilities are indeed shared across species.

We were struck for years, in assessing the literature on primate communication, by the fact that even some of the most fundamental empirical comparisons about vocal communication in humans and our close relatives had not yet been made: No quantification of the *amount* of vocalization and vocal interaction occurring at the beginning of life by humans and our closest phylogenetic relatives had ever been published, to our knowledge, prior to our own recent study[^1^](#_ENREF_1). The current work adds to the perspective: referencing the data figures in Oller et al.[^1^](#_ENREF_1), it can be seen that even the human preterm infants still in neonatal intensive care produce many times more protophones than protophone-like sounds produced by the three bonobo infants followed in the cited research. The rates of human preterm protophones proved also to be many times higher than protophone-like sounds in chimpanzee infants as estimated from work of Kojima[^43^](#_ENREF_43). Thus it seems near certain that our hominin ancestors were long under selection pressure to vocalize more freely and frequently than must have been the norm among other members of the ape family.

Still the pattern of results also implies that bonobo/chimpanzee vocal capabilities do indeed share foundations with the human ones. Laughter seems clearly to be homologous in humans and bonobos/chimpanzees, as others have argued[^44^](#_ENREF_44)^,^[^45^](#_ENREF_45). Some extremely negative expressions (e.g., screaming) are also likely homologous across the species, but our results suggest that acoustic patterns are divergent for such negative vocal expressions in infancy across humans and other apes.

In addition, although the protophones of the human infants even when still in the NICU were vastly more frequent in occurrence than potentially similar sounds in the bonobo infants, the data suggest a low rate of similar sounds does indeed exist in bonobos. Also, Kojima’s work with Pan and Pal[^46^](#_ENREF_46) suggests that rate of occurrence of protophone-like sounds may be sensitive to cultural entrainment, at least in chimpanzees. The lack of any maternal bonobo vocal elicitation or even mere vocalization directed at the infants in Oller et al.[^1^](#_ENREF_1) shocked us. We suspect, based on reports of others for both bonobos and chimpanzees, that such mother-to-infant vocalization does occur under some circumstances[^47^](#_ENREF_47), but that its base rate is very low. Again Kojima’s finding[^46^](#_ENREF_46) that Pan (who had been reared with vocally-eliciting humans) did indeed sometimes direct vocalization to her own daughter Pal, and that some vocal turn taking did occur between them, leaves the intriguing suggestion that the key ingredient in the apparent human evolutionary explosion of motivation to vocalize for fun and interaction may have been based on changes in the culture of hominins. The change we have in mind is based on hominin mothers increasingly vocalizing and eliciting vocalization from their infants, presumably in the attempt to obtain fitness information about them. Such information would have become increasingly relevant across evolutionary time to parental investment in the survival of their infants, who apparently showed an increasingly long period of helplessness (ultimately twice as long as in bonobos and chimpanzees) across hominin history[^48^](#_ENREF_48).

The plausibility of this scenario for the evolution of the earliest foundations of language, with its emphasis on cultural change in child-rearing, is also fortified by the fact that a wide variety of taxa have been shown able to learn a variety of foundational language capabilities, and even in some cases the rudiments of fully lexical items (sometimes only in comprehension, sometimes with hand signs, sometimes with picture-pointing symbology …), *if the animals have been human-reared and exposed to human language regularly*. These animals have included apes, dolphins, sea lions, dogs, and parrots, to name some of the most salient cases[^42^](#_ENREF_42). Critically all of these animals show essentially no language-like communication without the human training. The studies show that much of the infrastructure for language-like behavior is shared across many taxa, and that human-like vocal enculturation may be the key to stimulating activation of that infrastructure.

Furthermore recent results suggest that human evolution of exploratory and interactive vocalization as a mechanism of bonding may not have required terribly complex genetic changes. Evolved neuroanatomical linkages may have connected flexible corticostriatal learning with social motivational circuitry, creating a feedback loop between cortical control of the vocal system and social-reward circuitry, cementing a vocal interactive social bond out of which more complex structure could emerge[^49^](#_ENREF_49). Whatever the evolutionary changes were that led to human exploratory vocalization, they must have run deep indeed, given that even infants two months before due date already engage in such vocal exploration to a remarkable extent.

**S3: Audio examples (click on links)**

**From Figure 1, main text**

Fig1: 0m_Cry.WAV

Fig1: 0m_Growl.WAV

Fig1: 0m_Squeal.WAV

Fig1: 0m_Vocant.WAV

Fig1: 1m_Cry.WAV

Fig1: 1m_Growl.WAV

Fig1: 1m_Squeal.WAV

Fig1: 1m_Vocant.WAV

Fig1: minus1m_Cry.WAV

Fig1: minus1m_Growl.WAV

Fig1: minus1m_Squeal.WAV

Fig1: minus1m_Vocant.WAV

Fig1: minus2m_Cry.WAV

Fig1: minus2m_Growl.WAV

Fig1: minus2m_Squeal.WAV

Fig1: minus2m_Vocant.WAV

**From section S1.1.5**

FigS1_WailCry_Preterm_Minus1Month.wav

FigS2_Vocant_Preterm_Minus1Month.wav

FigS3_WailCry_Preterm_Minus1Month.wav

FigS4_Vocant_Fullterm_0months.wav

FigS5_WailCryWithCatchBreath_Fullterm_0months.wav

FigS6_Whimper_Fullterm_0months.wav

**References**

1 Oller, D. K. *et al.* Language origin seen in spontaneous and interactive vocal rate of human and bonobo infants. *Frontiers Psychology* **10**, doi:<https://doi.org/10.3389/fpsyg.2019.00729> (2019).

2 Caskey, M., Stephens, B., Tucker, R. & Vohr, B. R. Importance of Parent Talk on the Development of Preterm Infant Vocalizations. *Pediatrics* **128**, 910–916 (2011).

3 Zimmerman, F. *et al.* Teaching By Listening: The Importance of Adult-Child Conversations to Language Development. *Pediatrics* **124**, 342-349 (2009).

4 Xu, D., Richards, J. A. & Gilkerson, J. Automated analysis of child phonetic production using naturalistic recordings. *Journal of Speech, Language, & Hearing Research* **57**, 1638-1650, doi:doi:10.1044/2014 (2014).

5 Oller, D. K. *The Emergence of the Speech Capacity*. (Lawrence Erlbaum Associates, 2000).

6 Jhang, Y. & Oller, D. K. Emergence of Functional Flexibility in Infant Vocalizations of the First 3 Months. *Frontiers in Psychology* **8**, doi:doi.org/10.3389/fpsyg.2017.00300 (2017).

7 Oller, D. K. *et al.* Functional flexibility of infant vocalization and the emergence of language. *Proceedings of the National Academy of Sciences* **110**, 6318-6632, doi:doi: 10.1073/pnas.1300337110 (2013).

8 Yoo, H., Bowman, D. & Oller, D. K. The origin of protoconversation: An examination of caregiver responses to cry and speech-like vocalizations. *Frontiers in Psychology*, 1-15, doi:<https://doi.org/10.3389/fpsyg.2018.01510> (2018).

9 Jhang, Y., Franklin, B., Ramsdell, H. L. & Oller, D. K. Differing Roles of the Face and Voice in Early Human Communication: Roots of Language in Multimodal Expression. *Frontiers in Communication* **15**, doi:doi: <https://doi.org/10.3389/fcomm.2017.00010> (2017).

10 Yoo, H., Buder, E. H., Bowman, D. D., Bidelman, G. M. & Oller, D. K. Acoustic Correlates and Adult Perceptions of Distress in Infant Speech-Like Vocalizations and Cries. *Frontiers in Psychology* **10**, doi:10.3389/fpsyg.2019.01154 (2019).

11 Bosma, J. F., Truby, H. & Lind, J. Cry motions of the newborn infant. *Acta Paediatrica Scandinavia Supplement* **163**, 61-92 (1965).

12 Wasz-Hockert, Lind, J., Vuorenkoski, V., Partanen, T. & Valanne, E. *The Infant Cry: A Spectographic and Auditory Analysis*. (Heinemann, 1968).

13 Stark, R. E. & Nathanson, S. N. in *Fourth symposium on oral sensation and perception: development of the fetus and infant* (ed J. F. Bosma) 323-352 (US Govt. Printing Office, 1974).

14 Lynch, M. P., Oller, D. K., Steffens, M. L. & Buder, E. H. Phrasing in prelinguistic vocalizations. *Developmental Psychobiology* **28**, 3-23 (1995).

15 TF32 (University of Wisconsin- Madison, Madison, WI, 2001).

16 Oller, D. K. in *Child phonology, Vol 1: Production* (eds G. Yeni-Komshian, J. Kavanagh, & C. Ferguson) 93-112 (Academic Press, 1980).

17 Oller, D. K., Basinger, D. & Eilers, R. E. in *International Conference on Infant Studies.*

18 Oller, D. K. & Griebel, U. in *Evolution of Communicative Flexibility: Complexity, Creativity and Adaptability in Human and Animal Communication* (eds D. K. Oller & U. Griebel) 141-168 (MIT Press, 2008).

19 AACT (Action Analysis Coding and Training) (Intelligent Hearing Systems, Miami, FL, 2010).

20 Liang, K.-Y. & Zeger, S. Longitudinal data analysis using generalized linear models. *Biometrika* **73**, 13-22 (1986).

21 Stark, R. E. in *Child Phonology, vol. 1* (eds G. Yeni-Komshian, J. Kavanagh, & C. Ferguson) 73-90 (Academic Press, 1980).

22 Truby, H. M. & J, L. Cry sounds of the newborn infant. *Newborn infant cry: Acta Paediatrica Scandinavica* **163**, 7-59 (1965).

23 Lester, B. M. & Boukydis, C. F. Z. in *Nonverbal vocal communication* (eds H.Papoušek, U.Jürgens, & M.Papoušek) 145-173 (Cambridge University Press, 1992).

24 Locke, J. L. Parental selection of vocal behavior: Crying, cooing, babbling, and the evolution of language. *Human Nature* **17**, 155-168 (2006).

25 Furlow, F. B. Human neonatal cry quality as an honest signal of fitness. *Evolution and Human Behavior* **18**, 175-193 (1997).

26 Beebe, B., Alson, D., Jaffe, J., Feldstein, S. & Crown, C. Vocal congruence in mother-infant play. *Journal of Psycholinguistic Research* **17**, 245-259 (1988).

27 Gros-Louis, J., West, M. J., Goldstein, M. H. & King, A. P. Mothers provide differential feedback to infants' prelinguistic sounds. *International Journal of Behavioral Development* **30**, 509-516 (2006).

28 Trevarthen, C. & Marwick, H. in *Precursors of early speech* (eds B. Lindblom & R. Zetterstrom) 279-308 (Stockton Press, 1986).

29 Anderson, B. J., Vietze, P. & Dokecki, P. R. Reciprocity in vocal interactions of mothers and infants. *Child Development* **48**, 1676-1681 (1977).

30 Bloom, K. Patterning of infant vocal behavior. *Journal of Experimental Child Psychology* **23**, 367-377 (1977).

31 Jaffe, J., Beebe, B., Feldstein, S., Crown, C. L. & Jasnow, M. D. *Rhythms of dialogue in infancy: Coordinated timing in development*. Vol. 66(2) (Univ of Chicago Press, 2001).

32 Ginsburg, G. P. & Kilbourne, B. K. Emergence of vocal alternation in mother-infant interchanges. *Journal of Child Language* **15**, 221-235 (1988).

33 Jasnow, M. & Feldstein, S. Adult-like temporal characteristics of mother-infant vocal interactions. *Child Development* **57**, 754-761 (1986).

34 Hilbrink, E. E., Gattis, M. & Levinson, S. C. Early developmental changes in the timing of turn-taking: a longitudinal study of mother–infant interaction. *Frontiers in Psychology* **6**, doi:<http://dx.doi.org/10.3389/fpsyg.2015.01492> (2015).

35 Caskey, M., Stephens, B., Tucker, R. & Vohr, B. R. Adult Talk in the NICU With Preterm Infants and Developmental Outcomes. *Pediatrics* **133**, e578–e584 (2014).

36 Bickerton, D. *Language and Species*. (University of Chicago Press, 1990).

37 Cangelosi, A. & Parisi, D. in *Simulating language evolution* (eds A Cangelosi & D. Parisi) 3-28 (Springer-Verlag, 2002).

38 Deacon, T. W. *The Symbolic Species*. (W. W. Norton & Co. Ltd., 1997).

39 Jackendoff, R. & Pinker, S. The nature of the language faculty and its implications for evolution of language (Reply to Fitch, Hauser, and Chomsky). *Cognition* **97**, 211–225 (2005).

40 Niyogi, P. *The Computational Nature of Language Learning and Evolution*. (MIT Press, 2006).

41 Sinha, C. in *The Evolution of Communication Systems: A Comparative Approach* (eds D. K. Oller & U. Griebel) 217-235 (MIT Press, 2004).

42 Griebel, U., Pepperberg, I. M. & Oller, D. K. Developmental plasticity and language: A comparative perspective. *Topics in Cognitive Science (topiCS)* **8**, 435-445 (2016).

43 Kojima, S. *A Search for the Origins of Human Speech*. (Kyoto University Press, 2003).

44 Davila Ross, M., Owren, M. J. & Zimmermann, E. Reconstructing the evolution of laughter in great apes and humans. *Current Biology* **19**, 1106-1111, doi:doi:10.1016/j.cub.2009.05.028 (2009).

45 Davila Ross, M., Owren, M. J. & Zimmermann, E. The evolution of laughter in great apes and humans. *Communicative & Integrative Biology* **3**, 191-194 (2010).

46 Kojima, S. in *Primate origins of human cognition and behavior* (ed Tetsuro Matsuzawa) 190-196 (Springer-Verlag Publishing, 2001).

47 Bermejo, M. & Omedes, A. Preliminary vocal repertoire and vocal communication of wild bonobos (Pan paniscus) at Lilungu (Democratic Republic of Congo). *Folia Primatologica* **70**, 328-357 (1999).

48 Locke, J. L. & Bogin, B. Language and life history: A new perspective on the evolution and development of linguistic communication. *Behavioral & Brain Sciences* **29**, 259-325 (2006).

49 Syal, S. & Finlay, B. L. Thinking outside the cortex: Social motivation in the evolution and development of language. *Developmental Science* **14**, 417-430, doi:DOI: 10.1111/j.1467-7687.2010.00997.x (2011).
